# Computational Performance of Deep Reinforcement Learning to Find Nash Equilibria

**DOI:** 10.1007/s10614-022-10351-6

**Published:** 2023-01-03

**Authors:** Christoph Graf, Viktor Zobernig, Johannes Schmidt, Claude Klöckl

**Affiliations:** 1 Institute for Policy Integrity, New York University, New York, NY 10012 USA; 2 Program on Energy and Sustainable Development (PESD), Stanford University, Stanford, CA 94305-6072 USA; 3 Institute for Sustainable Economic Development, University of Natural Resources and Life Sciences, Vienna, Austria

**Keywords:** Bertrand equilibrium, Competition in uniform price auctions, Deep deterministic policy gradient algorithm, DDPG, Parameter sensitivity analysis

## Abstract

We test the performance of deep deterministic policy gradient—a deep reinforcement learning algorithm, able to handle continuous state and action spaces—to find Nash equilibria in a setting where firms compete in offer prices through a uniform price auction. These algorithms are typically considered “model-free” although a large set of parameters is utilized by the algorithm. These parameters may include learning rates, memory buffers, state space dimensioning, normalizations, or noise decay rates, and the purpose of this work is to systematically test the effect of these parameter configurations on convergence to the analytically derived Bertrand equilibrium. We find parameter choices that can reach convergence rates of up to 99%. We show that the algorithm also converges in more complex settings with multiple players and different cost structures. Its reliable convergence may make the method a useful tool to studying strategic behavior of firms even in more complex settings.

## Introduction

A fundamental challenge in many applications in economics and social sciences is to derive counterfactual outcomes of the world—a necessity to, e.g., analyze the impact of a policy intervention. For many applications, this task is challenging because of the lack of measurements on certain important variables, potential measurement errors, or because the underlying mechanisms are complex and poorly understood. If the exact mechanism of how the world operates is unknown, experiments or causal statistical models, both in combination with randomization, may be successfully deployed to derive valid counterfactual outcomes. There exists, however, a subset of economic problems in which the mechanisms are precisely defined, e.g., auctions. While the auction platform is typically set up centrally and operates based on a pre-determined set of rules, participants engage with each other “through” the auction mechanism. The auction clearing for given offers and bids from participants is typically trivial to solve; this is, however, usually not true for the derivation of market participants’ strategies.^1^

If large comprehensive datasets were available, counterfactual outcomes of auctions could be evaluated using non-parametric structural estimation techniques (see, e.g., Guerre et al., 2000; Kastl, 2011; Caoui, 2022; Charankevich, 2021). Alternatively, game-theoretic models could be deployed to derive counterfactual outcomes. However, in practice these models may be computationally challenging to solve because of their two-stage nature. More precisely, firms competing in an auction rely on the outcome of the second stage, i.e., the auction clearing.^2^ A relatively new approach, that we put to the test in this paper, is to use reinforcement learning to model strategic interactions in auction-based markets.

In this framework, market participants or players interact through the environment, i.e., the auction clearing mechanism, with each other using a combination of experimenting and learning. One advantage is that this approach does not require any empirical data on behavior of market participants; it only requires the knowledge of how the auction is organized (market rules) and a description of the agents’ objective functions as well as their (physical) capacity limits and costs of production. The “data” is then constructed on the fly as a combination of the strategies generated by the players and the corresponding auction clearing results. This procedure is related to agent-based models from the field of computational economics (Tesfatsion & Judd, 2006) where computational models are applied to less understood areas (e.g., out-of-equilibrium dynamics). A frequent critique of agent-based models is that outcomes are driven by the assumptions on how agents are designed (see, e.g., Hommes, 2006; Deissenberg et al., 2008, who model agents using predetermined decision rules). The fundamental difference in the reinforcement learning setting is that only mild assumptions on the agent’s behavior are encoded, for example, their intention to maximize their profits given their available production capacity and production costs. Behaviour in a certain auction environment is then informed through a combination of randomly exploring the action space and learning from past experience which actions performed well under which circumstances. Hence, in reinforcement learning an agent’s behavior is not predefined but arises emergently from the interplay of environment, competing agents, and the reward mechanism.

The purpose of this paper is to apply deep reinforcement learning to learn strategic offer behavior in a price competition environment and more generally in uniform price auctions where firms are capacity constrained. Specifically, we employ the deep deterministic policy gradient (DDPG) algorithm (Lillicrap et al., 2015) to learn an agent’s behavior. This algorithm is capable to tackle fully continuous state and action spaces. Furthermore, this and similar recent deep reinforcement learning algorithms have been successfully deployed to master strategic games such as Poker (Brown et al., 2019), Go (Silver et al., 2016; Schrittwieser et al., 2020), or Starcraft (Vinyals et al., 2019), and it appears that these algorithms consistently outperform humans in playing these games. However, to the best of our knowledge, the current wave of deep learning algorithms has not yet been applied to study strategic behavior in auctions-based market such as e.g., wholesale electricity markets, and we are confident that, given the algorithm’s performance in (computer) gaming, it will improve on other heuristic methods such as classical *Q*-learning (Calvano et al., 2020; Asker et al., 2021), genetic algorithms (Andreoni & Miller, 1995; Noe et al., 2012), or particle swarm optimization (Boyer & Brorsen, 2014). As pointed out in Noe et al. (2012), genetic algorithms exhibit difficulties to reproduce standard Bertrand competition in 1st and 2nd price auctions. Generally, it has been observed that 2nd price auctions are a challenging environment for self-learning agents (Andreoni & Miller, 1995). The limited feedback on payoffs via experimentation as discussed in Harrison (1989) or Merlo and Schotter (1992) has been named as one reason. More precisely, a bidder’s best-response function often has a “flat maximum” because the cost of deviating from equilibrium strategies is relatively small (Noe et al., 2012). Neural networks play a central role in DDPG. These may lead to smoother best-response functions and allow to store past behavior in an approximated yet efficient way, which may help to successfully identify equilibria. Furthermore, highly efficient computational libraries are readily available, which allows conducting thousands of iterations of learning for a wide range of meta-parameters, thus enabling an understanding of if and under which conditions DDPG is able to identify Nash equilibria.

It is well-known that deep learning algorithms, to realize their full potential in any given problem domain, rely on a proper choice of learning parameters. Therefore, this work aims to facilitate the understanding of the parameter settings critical for the performance of DDPG in our environment. We focus on a relatively simple economic problem that has an analytical solution in the static case which we use as a benchmark. More precisely, we analyze the effect of learning parameter settings on outcomes in a simple symmetric unconstrained Bertrand duopoly, i.e., where two firms compete in prices for a fixed level of demand. The Bertrand model can be analyzed in a one-stage setting following the argument that both firms would compete each other down to their marginal cost. However, it could also be considered in a two-stage setting where the second stage is the market clearing of a uniform price auction following the first stage in which firms submit their offers. Because both firms are assumed to have unlimited capacity, auction clearing will award the low-offering firm to serve all of the demand.^3^ The advantage of this setting is that it paves the way for more realistic cases, for example, when firms are capacity constrained as in our second case study. Needless to say, more sophisticated modeling assumptions, for example, accounting for firms’ production portfolios (multi-unit auctions), sequential market interactions,^4^ or including dynamic constraints which may be relevant in wholesale electricity market settings where conventional generators have non-convex production functions,^5^ are relatively easy to integrate into this framework. However, the purpose of this paper is to first understand the effect of the parameter settings in the reinforcement learning framework on outcomes. Therefore, we constrain ourselves at this time to study the two simple cases which we can compare to analytically derived equilibrium outcomes.

Our main findings are that (i) DDPG is a reliable tool to study competition through auctions in both capacity constrained and unconstrained settings, (ii) DDPG improves upon established *Q*-learning and policy gradient methods because it does not require discretization or strong behavioral assumptions, (iii) convergence rates can reach up to 99% given optimal parametrization, (iv) convergence depends critically on choice of memory buffer size and noise decay rate for the unconstrained case, (v) convergence depends critically on choice of normalization method, learning rates and memory model in the capacity constrained case, (vi) both the choice of normalization method and memory model impact the eagerness of agents to engage in competition and hence have to be seen as modelling choices, even if not imposing strict decision rules. Our algorithm is open-source and accessible publicly through our Github repository.^6^

Calvano et al. (2020) and Asker et al. (2021) are two recent contributions that pursue a similar goal, that is, to evaluate the effect of parameter settings on equilibrium outcomes in reinforcement learning methods. Both articles deploy *Q*-learning (Watkins, 1989) methods which rely on a discrete state and action space. However, because all the outputs of different input (space, action) combinations must be stored in a so-called *Q*-table, discretization will always be coarse in practice given the limitations of computational resources. Instead, we use a deep-learning framework that allows us to approximate the *Q*-table and therefore enables us to work on a continuous state and action space. Moreover, scaling to larger numbers of features and players will not be possible for methods that exhibit exponential scaling in the number of features, while deep reinforcement learning has been shown to work in environments with high-dimensional state and action spaces and a high number of players. An interesting comparison to our work in the electricity market context is Lago et al. (2021), who deploy an approximate algorithm called fitted *Q*-iteration. Albeit not a deep reinforcement learning approach in the strict sense, they use a similar approximation strategy that seems to allow them to use more complex state spaces.

The remainder of the paper is structured as follows. In Sect. 2, we provide a brief exposition of the established methods to represent strategic interactions including the discussion of analytical and computational methods. In Sect. 3, we give an in-depth discussion of reinforcement learning’s recent developments and the recent paradigmatic shift towards deep reinforcement learning. We discuss both classical reinforcement learning, exemplified by *Q*-Learning, and deep reinforcement learning, exemplified by DDPG. In Sect. 4, we start with an analytical analysis of our main benchmark scenario, where we derive its range of static Nash equilibria. Our benchmark scenario contains both the capacity constrained and unconstrained cases. We go on in Sect. 5 to evaluate DDPG’s performance relative to its known analytical solution. We accompany convergence results, with a thorough investigation of the involved learning parameters and variational analysis of the parameters. We give recommendation on choices of learning rates, memory, normalization, memory buffer size and noise decay rates. We wrap up our results in Sect. 6.

## How to Model Strategic Interactions? From Game-Theoretic Equilibrium Models to Reinforcement Learning

Game theory (GT) aims to predict outcomes where players interact strategically with each other. The theory’s scope is wide ranging from economic applications, over biology to computer science. Nonetheless, GT originated from the systematic study of various card and board games such as Poker and Chess. In a strikingly similar development, competitively playing algorithms for computer strategy games are one of the hallmark successes of reinforcement learning. The similarities are strengthened by some theoretical works that reformulate training problems into specific types of games (Schuurmans & Zinkevich, 2016). This leads us to conclude that both fields have significant overlap despite there completely different methodological approach, thus making their comparison and interesting field of study. In the following, we briefly review key ideas of the two distinct fields and aim to asses how both fields can complement each other.

### From Game Theory ...

In order to predict outcomes in a strategic interaction among players, GT relies on the notion of equilibrium. A game with a unique pure strategy equilibrium is regarded as strong evidence that players will eventually reach this outcome. An equilibrium is constituted of strategies that are mutual best responses to each other, i.e., no player has an incentive to marginally deviate from her strategy given that the other players are playing their equilibrium strategies. In simple games, finding pure strategy equilibria involves typically a two-step procedure: (i) derive best responses of each player, and (ii) find set of overlapping best responses if they exist. More sophisticated games may be solved analytically (e.g., by deriving best response functions and finding their interaction) or they can be solved numerically involving non-convex optimization techniques (see, e.g., Graf & Wolak, 2020). Best responses always exist but they are not necessarily unique and in fact best response functions can be complicated objects, that do not necessarily allow to derive unique pure strategy equilibria.

The hardness of deriving equilibria depends strongly on the specificities of a game’s formulation. Generally, derivable equilibria tend to be relatively common in single-round games. For instance, a single round of Cournot competition or Bertrand competition with differentiated products allow non-trivial closed form solutions if the functional forms defining costs and demand are favorable. However, adding dynamics to the one-shot game poses significant challenges. In infinite games or finite games with uncertain termination point meta-strategies, such as e.g., punishments of non-cooperators—a strategy which would not make sense in a one-shot game—arise. The issue is even further complicated by the fact that the efficiency of such meta-strategies depends critically on model parameters such as the discount rate which in practice is hard to measure. A whole class of theorems, so-called “folk-theorems,” derive results that can essentially enforce any of a game’s static equilibria as an equilibrium of the entire extended game depending on the value of the discount rate (Fudenberg & Maskin, 1986).

In fact, only highly stylized cases support unique equilibria for extended games. More commonly, we face situations with either infinitely many equilibria or no pure strategy equilibrium at all. Furthermore, for mixed equilibria existence results are known, but not necessarily a constructive way to compute them. The lack of constructive equilibria for repeated games is an unsolved problem within GT. Due to the abundance of equilibria as predicted by folk theorems, this is strongly related to the question of equilibrium selection. Consequently, many heuristic approaches for equilibrium selection exist. Furthermore, refinements of the Nash equilibrium concept, such as e.g., the requirement of sub-game perfection, are often used to reduce the number of equilibria. Alas, in the frequent case of non-unique equilibria, there is no unified theory of equilibria discrimination that allows to decide which equilibria are more likely or advisable to be played. A systematic resolution of the issue does not seem imminent and is one of the key motivations for us to look beyond game theory towards solution concepts from outside of game theory in order to model mid- to long-term strategic interactions.

Computational methods may very-well complement analytical methods in economic settings similarly to the field of evolutionary game theory, where strong analytical results are available for its standard setting, i.e., mixed infinitely large populations, strong selection, and weak mutation (Fudenberg & Levine, 1998). However, these results frequently do not hold if those assumptions are violated and stochastic learning models are used increasingly to explore behavior beyond evolutionary game theory’s standard assumptions (Adami et al., 2016).

### ... over Algorithmic Game Theory ...

Classic Game Theory typically concerns itself with analytical derivations, existence, and uniqueness results of Nash equilibria. It usually does not deliver a theory of suboptimal and out-of-equilibrium play. Out-of-equilibrium play occurs prior to all players arriving in a given equilibrium and external influences may require frequent “re-equilibrations.” GT usually does not address whether the time scales spent in such an out-of-equilibrium play are short or long. Algorithmic game theory (AGT) addresses this issue by analyzing whether a given Nash equilibrium can be computed in finite or even short time scales (Roughgarden, 2010).

For instance, this is already illustrated by algorithmic game theory’s analytical results that particular learning behavior assumptions are able to efficiently learn specific subsets of equilibria (i.e., best response-dynamics converge to correlated equilibria, see e.g., Foster & Vohra, 1997; Awerbuch et al., 2008) and no-regret-dynamics converge to coarse correlated equilibria (Blum et al., 2008). Moreover, there are results that even Nash equilibria can be found efficiently for specific types of games such as potential games (Viossat & Zapechelnyuk, 2013). Thus AGT adds the important quality criterion of computational efficiency to GT’s toolbox.

However, algorithmic game theory’s results stems from theoretical computer science with a complexity theoretic flavour, where frequently theoretical algorithms are used to reason about existence and no-go results. This means that AGT’s notion of computational efficiency usually equals the complexity theoretic notion of polynomial time computable (Roughgarden, 2010). AGT’s algorithms are not necessarily always practically efficient or even constructive. As such, AGT’s approach refines the fixed-point theorem approach, but offers constructive results only in particular cases.

Furthermore, we are not aware of comparably strong results for bidding games as we have at our disposal in the case of potential games. We therefore hope to supplement these results with the use of concrete state-of-the-art deep learning algorithms that are considered practically successful in the Reinforcement Learning community and critically asses their viability for learning equilibrium strategies.

### ... to Reinforcement Learning

GT relies on the concept of a Nash equilibrium to predict a given player’s behavior. An alternative solution concept could be to simulate a players behaviour by providing a learning algorithm that extrapolates from past rounds to future rounds and recommends the next move. This move is not necessarily an optimal move, but it is the move that appears optimal given a certain set of experiences and learning parameters.

GT relies on mathematical optimization and the theory of metric spaces to identify mutual best responses, i.e., equilibria. Reinforcement learning (RL) essentially is a form of educated Monte-Carlo simulation, where randomly simulated moves are used as a basis for a statistical inference that determines what strategy is the best conditioned on a player’s history.

There exists a certain trade-off between both approaches here. More precisely, reinforcement learning algorithms can by design give no guarantee or certificate of optimality. However, reinforcement learning is versatile and does not require specific functional forms, like convexity, of the constraints or objective function, that are common in mathematical optimization. Learning algorithms can either converge naturally or convergence can be enforced through the choice of hyper-parameters. The quality of such solutions may vary, especially if convergence is enforced. Nevertheless, once convergence is attained, a strategy is always singled out.

Our main conclusion is that DDPG can find Nash equilibria in our benchmark scenario. Therefore, DDPG is a useful algorithm in the price competition game: first, we want to highlight that algorithmic-trading has become a commodity in electricity market trading (see, e.g., Lehna et al., 2022, and literature references therein), and we believe that DDPG could be a viable candidate to be deployed in that domain. Therefore some of the actions performed on real markets may follow DDPG’s logic in the future. Second, other authors find indeed agreement between NE and human behaviour in energy market games. Thurber et al. (2015) find experimental evidence that suppliers which have the chance of being pivotal, offer their capacity at the price cap. Furthermore, the authors find that in a setting where suppliers do not perceive themselves as being able to influence the market clearing price with their offers, marginal cost offering by all suppliers prevails. This always coincides with the DDPG solution for the high-offering player who offers at the price cap, while the low-offering player typically offers in a limited range including marginal costs but also a certain interval above. It has to be noted, that this range coincides with the theoretical predictions.

## Reinforcement Learning: From the Q-table to Deep Neural Networks

We devote the following section to a discussion of recent advances in the machine learning domain with a focus on algorithms that we deem best suited to learn strategic bidding in uniform price auctions.

Machine learning is subdivided in supervised, unsupervised, and reinforcement learning. Unsupervised learning is completely independent of the problem structure making it versatile but also somewhat limited in scope. It is usually used for classification or prediction tasks. Supervised learning in-turn relies on human experts to train algorithms. In supervised learning, experts discriminate exemplary “training” actions into good or bad. The algorithm then learns to mimic the provided discrimination into good and bad and is as such not independent of the modellers input. In our opinion, the third option, RL, is the most interesting approach for tackling strategic games. It relies on an exact specification of an reward mechanism, that trains players that seek to increase their respective reward. In many markets that rely on auctions—electricity markets are no exception to that—the market clearing mechanism is known. That means, for an exogenous set of offer and bid curves for each market participant, the market clearing results can be precisely computed. The challenge though, is to derive how market participants will formulate the offer curves they submit to the market. Sometimes RL agents are modelled as choosing from a set of pre-specified offer-curves (Spooner et al., 2018; Lussange et al., 2021), but remember that RL algorithms, in principle, require only to specify, i.e., hard-code, the reward mechanism. Given that, equilibrium offer and bid curves may be learned by iteratively submitting curves to the market clearing mechanism and receiving a response, i.e., a per-round profit value conditional on my submitted curve and the curves submitted by my opponents.

Moreover, Sirignano and Cont (2019)—similarly to our work—address economic competition using a continuous deep learning framework. Specifically, Sirignano and Cont (2019) propose a supervised learning algorithm that predicts stock markets.

### Reinforcement Learning

We briefly review reinforcement learning’s key concepts (see, e.g., Sutton & Barto, 2018, for additional details on the concept).

Reinforcement learning’s goal is to determine an optimal action given a certain state, while drawing only on the information of a sequence of past rewards. In order to capture the inherent time-structure of RL, we define a time index *t*. Let us write the action at time *t* as $a_{t}$, the state at time *t* as $s_{t}$, and the reward received at time *t* by $R(a_{t},s_{t})$ depending on $a_{t}$ and $s_{t}$. We do not specify *a* and *s* closer by intention, since their exact meaning depends on the specific problem. However, we point out that they are not necessarily integers or even single numbers, they could be vectors or chosen from an continuous interval. Similarly, infinite time sequential optimization tries to maximize a value

1 $$\begin{aligned} V(s_{0}):= \sum _{t=0}^{\infty }\max _{a_{t}} \gamma ^{t} R(a_{t},s_{t}) \end{aligned}$$

that is essentially a time averaged discounted objective function, where $0 \le \gamma < 1$ is a discount factor required to enforce (1)’s convergence. In infinite time sequential optimization, there are various tools such as dynamic programming to explicitly evaluate (1) if possible. Nonetheless, this can be a non-trivial task. RL shares the same aim as the field of mathematical optimization, but can be considered an alternative methodological approach. Hence, the goal of RL is to converge towards

2 $$\begin{aligned} \mathop {\textrm{argmax}}\limits _{a} R(a,s), \end{aligned}$$

for all *s* or at least approximately find $a_{t}$ such that

3 $$\begin{aligned} \sum _{t=0}^{\infty }\gamma ^{t} R(a_{t},s_{t}) \approx V(s_{0}). \end{aligned}$$

In RL an action is determined from a sequence of rewards and actions $a_{0}, \ldots ,a_{t}$ taken in specific states $s_{0}, \ldots ,s_{t}$ by means of a learning rule. Sequential optimization problems can be decomposed by means of the (infinite) Bellman equation as

4 $$\begin{aligned} V(s_{0}):= & {} \sum _{t=0}^{\infty }\mathop {\textrm{argmax}}\limits _{a_{t}} \gamma ^{t} R(a_{t},s_{t})= R(a_{0},s_{0}) + \sum _{t=1}^{\infty }\mathop {\textrm{argmax}}\limits _{a_{t}} \gamma ^{t} R(a_{t},s_{t}) \nonumber \\= & {} R(a_{0},s_{0}) + \gamma V(s_{1}). \end{aligned}$$

Solving (4) involves several well-known challenges. First, infinite time horizon problems do require a so-called discount factor $\gamma$ to become solvable. The important modelling choice of $\gamma$ is common to all approaches relying on (4) and not limited to RL at all. Similarly, game-theoretic folk theorems (Fudenberg & Maskin, 1986) encounter the problem of solutions relying sensitively on the choice of $\gamma$. Second, if no closed form solution is known for (4), computationally solving an infinite expression is not feasible. Therefore, it is common practice to solve relaxed versions of (4) to obtain approximately optimal solutions. In essence, all reinforcement learning methods are specifications how to approximate $V(s_t)$ in (4) from a given history of *t* actions and states.

### Q-Learning

The main model-free approach to estimate *V* is the family of temporal difference learning algorithms. Its most well-known member is so-called Q-learning (Watkins, 1989). *Q*-learning’s core assumption is that the state action space is discrete and that the discounted cumulative reward function $V(s_{t})$ can thus be represented as a table or matrix whose values contain the possible rewards originating $R(a_{t},s_{t})$ from any combination of *S* states and *A* actions. If such a table were available, finding optimal actions for a state reduces to reading of the maximal value in a column corresponding to the state of interest.

*Q*-learning’s key strategy is to find a matrix representation of $V(s_{t})$ through an update rule

5 $$\begin{aligned} Q_{t+1}(s_t,a_t) = (1-\alpha ) Q_t(s_t,a_t) + \alpha [R(s_t,a_t) +\gamma \max _{a}Q_t(s_{t+1}, a)], \end{aligned}$$

where *Q* is an initially arbitrary matrix of size $S \times A$, $\alpha$ is the so-called learning rate and $\gamma$ the discount factor. Repeated application of (5) will eventually lead to convergence towards $R(s_t,a_t) +\gamma \max _{a_t}Q_t(s_{t+1}, a_t)$ if *R* remains stationary and all values of $Q_t(s_t,a_t)$ are updated alike. $R(s_t,a_t) +\gamma \max _{a_t}Q_t(s_{t+1}, a_t)$ is the first order approximation of the Bellman equation (4), hence under the assumptions of stationarity, ergodicity, and sufficiently many iterations, the matrix *Q* is expected to converge towards an approximation of *V*.

*Q*-learning usually selects the action that has the maximal value in the *Q*-table, but ergodicity is only ensured by sometimes playing a random move instead (chosen with probability $\epsilon$ the exploitation-exploration parameter). Typically, $\epsilon$ is decreased throughout the run-time of the algorithm until it reaches a lower threshold. This allows for easier convergence after many iterations. The speed of the decrease in $\epsilon$ is termed the decay rate.

Under ideal conditions, *Q*-learning can thus reliably approximate the Bellman equation. However, *Q*-learning’s fundamental prerequisites can be hard to ensure in practice. For instance, stationarity of rewards is a strong assumption in general, but also particularly when studying auctions. *Q*-learning is traditionally used by single agents. Especially, in the multi-agent case relevant for auctions, competing agents essentially form a dynamic state space that makes it hard to ensure stationarity (Bu et al., 2008; Foerster et al., 2016). In addition, it becomes more difficult to attain ergodicity and to achieve short running times as the state-action space becomes larger. This means that fine discretizations of the state space leads to unfavourable scaling in the number of features and can lead to humongous state-action spaces even in relatively simple models. Furthermore, memory requirements constrain modelers to use rather coarse model descriptions in practice (see Table 1). Aside from scaling considerations, we want to close this sections with a cautionary tale stemming from theoretical considerations in Operations Research. It is self-evident that discretization of state spaces introduces rounding errors. Naturally, a discrete and continuous algorithm will be operating in slightly different scenarios due to their respective action spaces. Hence, *Q*-learning will in-fact play a slightly distorted discretized version of the more general continuous game. The fineness of the discretization directly influences the number of ensuing equilibria. For instance, even straight-forward discretized Bertrand games are examples that exhibit 2–3 equilibria depending on the choice of discretization, while the continuous Bertrand game is actually unique (Asker et al., 2021).

Sometimes discretization is justified with the perceived negligibility of small rounding errors. Nonetheless, discretization alters the underlying problem fundamentally. For instance, a discretized linear program becomes an integer linear problem whose solution can be arbitrary far away from the originally continuous problem, regardless of the possibly small magnitude of the rounding errors (see IP Myth 1 in Greenberg, 2010). Hence, discretization is not necessarily a stable operation and can alter a problem’s solution which may bias *Q*-learning’s outcomes.

**Table 1:** Comparison of recent articles applying *Q*-learning

	Viehmann et al. (2021)	Calvano et al. (2020)	Asker et al. (2021)	Aliabadi et al. (2017)	Lussange et al. (2021)^b^	Spooner et al. (2018)
Domain	Sequential electricity markets (multi-unit auctions)	Bertrand duopoly	Bertrand oligopoly	Electricity markets (multi-unit auctions)	Stockmarket	Stockmarket
Action space scope	Single price-offer per unit	Single price-offer	Single price-offer	Single price-offer	Volatility and value forecast (critic), predefined bidding strategies (actor)	Predefined bidding strategies
Action bounds	$\left[ 0, 10 \right] ^{2}$	Bertrand- to monopoly prize	$\left[ 0.1, 10 \right]$	$\left[ 9, 45 \right]$	$\left[ 0, 2\right] ^{3}$ (critic) $\left[ 0, 2\right] ^{2}$ (actor)	$\left[ 0, 9\right]$
Action step-size	1	Scale-dependent	0.1	9–10	[low, mid, high]^3^	9 strategies
Action space-size	$10^2$	15	$10^{2}$	$3\cdot 4\cdot 4=48$	$3^3$ (critic) $3^2$ (actor)	10
State space scope	Marginal price, volume weighted average price, and total demand^a^	Single round memory	None	None	Past value and variances (critic) Stockmarket indicators (actor)	Stockmarket indicators
State step-size	1	Scale-dependent	None	None	[Low, mid, high]^c^	Tile code
State space size	$10^2$ ($\cdot$ demand)	$15^2$	1	1	$3^3$ (critic), $3^3 \cdot 2^2$ (actor)	32
State-action space size	$10^4$	$15^{3}$	$10^2$	48	$3^9=729$ (critic), $3^3 \cdot 2^2 \cdot 3^3 \approx 8 \cdot 10^5$ (actor)	320

^a^Demand is only used in few scenarios with unspecified granularity. Graphs show possible granularities of 5 or 100.

^b^Lussange et al. (2021) employs a discretized Actor-Critic design, with a Forecast (i.e., Critic) and a Trade (i.e., Actor) algorithm interacting together. The tables lists both state and action spaces.

^c^All actions, states, forecasts and bid-ask prices are aggregated from time series into a low, mid, and high classification. Exact prices are then computed by predefined rules

Table 1 gives a non-exhaustive overview of very recent reinforcement learning implementations of multi-agent reinforcement learning in economic settings. Table 1 compares the relation between a paper’s area of study and the granularity of state and action representation. *Domain* summarizes what scenario the paper describes. We contrast algorithms for competition in abstract oligopolies, electricity markets and stock markets. *Action and state space scope* details how states and actions are modelled. Action spaces are either defined as (i) choosing predefined strategies that directly compute bid prices from averages, minima and maxima of the latest observed prices (Spooner et al., 2018; Lussange et al., 2021), or by (ii) picking from a discrete scale of prices (Aliabadi et al., 2017; Calvano et al., 2020; Viehmann et al., 2021; Asker et al., 2021). While (i) is a behavioral restriction and hence not model-free, (ii) could in principle be model free. However, the action spaces tend to be relatively constrained both in range and granularity with action spaces including 10–100 distinct actions, that may equally lead to a behavioral restriction of the agents. Naturally, modelling choices have to be made. We describe those both for action and state spaces with respect *bounds, space-size, and step-size*. Bounds denote upper and lower limit of the modeled space. Space-size is the number of elements in the space. Finally, step-size denotes the distance between individual elements. We employ the convention, that we write a given state’s size as product of its individual constituents (i.e., $10 \times 10=10^{2}$ labels a space containing two distinct features with 10 possible values each). Whether a given number of state-action pairs appears sufficient, depends on the desired accuracy and modelling domain. The employed state-action spaces tend to reside in the order of $10^{2}$–$10^{5}$ (Calvano et al., 2020; Viehmann et al., 2021). Despite the relatively large magnitude of the composite state-action space, the resolution of individual features remains relatively modest typically ranging from 1 to 10 units, unless trivial state spaces are involved. The number of distinct features (i.e., actions or state space elements) usually is less than 3. However, even slightly older papers can be found that exhibit action spaces of less then 100 elements (Aliabadi et al., 2017).

Overall, we have discussed several challenges experienced within classical reinforcement learning exemplified by *Q*-learning. Not all of these challenges can be expected to be easily overcome by deep learning. For instance, non-stationary state spaces are a recognized problem of the notoriously hard multi-agent games that seem unavoidable from an algorithmic perspective and remain common to all reinforcement learning methods, be it classical or deep.

However, other issues can very well be addressed by deep learning. Consider, either *Q*-learning’s problematic state-action space scaling or its possible discretization errors. Both are intimately tied to *Q*-learning’s natural discreteness and can be remedied through the application of deep learning methods. Although there exist deep learning approaches for discrete problems as well (for instance, DQN, Mnih et al., 2015) or Lussange et al., 2021), we will go on to elaborate on the possibilities of natively continuous algorithms in the following section.

### Deep Deterministic Policy Gradient

Deep Deterministic Policy Gradient (DDPG) extends *Q*-learning by two key concepts: *neural networks* and *actor-critic* design.

First, when opting for a neural network based design, we forfeit the idea to explicitly storing information about any possible state action combination. This is desirable whenever the state-action space grows larger. Some problems may be well-representable with coarse discretizations and these are exactly those where *Q*-learning will perform well. Alas, many naturally occurring quantities such as temperature or quantities of energy are inherently continuous parameters. Even essentially discrete parameters such as prices may require very fine discretizations if adressed in full-generality (i.e., down to cent scale). For such problem domains, neural network based designs allow to represent continuous as well as almost continuous domains easily.

If $Q(s_{t}, a_{t})$ grows too large to memorize and process efficiently, one could be satisfied with finding a parametrized family of functions that guarantees

6 $$\begin{aligned} \min _{\omega _{1},\ldots ,\omega _{p}} \sum _{t}||C(\omega _{1},\ldots ,\omega _{p},s_{t},a_{t}) - Q(s_{t},a_{t})||_{2} < \delta , \end{aligned}$$

for a non-linear function *C*, a sufficiently small $\delta$ and a fixed set of parameters $\omega _{1},\ldots ,\omega _{p}$, where we require $p \ll S \cdot A$ (i.e., there are significantly less weights required, than needed for an exhaustive state space description). We call this neural network *C* the *Critic* network and $\omega _{1},\ldots ,\omega _{p}$ its *weights*. (6) states that *C* is an approximation of the *Q*-table *Q*, that is in turn an approximation of the problems discounted cumulative reward function *V* obtained from the Bellman equation. This would allow us to store comparable amounts of information with much less parameters. In principle, many function families can attain this, however neural networks have been proven to always fulfill (6) if *p* is large enough by the so-called universal approximation theorem (Cybenko, 1989).

This allows us to make the approximation

7 $$\begin{aligned}{} & {} (1-\alpha ) Q(s_t,a_t) + \alpha [R(s_t,a_t) +\gamma \max _{a}Q(s_{t+1}, a)] \nonumber \\{} & {} \approx (1-\alpha ) C(\omega _{1},\ldots ,\omega _{p},s_t,a_t) + \alpha [R(s_t,a_t) +\gamma \sup _{a}C(\omega _{1},\ldots ,\omega _{p},s_{t+1}, a)], \end{aligned}$$

where we do only memorize a finite number of parameters $\omega _{1},\ldots ,\omega _{p}$ and not necessarily the possibly many $a_{t}$ and $s_{t}$ arising in complex state-action spaces. Moreover, a function allows for continuous outputs, thus paving the way for continuous state-action spaces.^7^

However, a further complication arises when moving to continuous action spaces. The evaluation of (7)’s left hand side is straight forward. In particular, it is easy to evaluate the maximum $\max _{a}Q(s_{t+1}, a)$ since *Q* is just a finite table. In *Q* each row (or columns) is simply a finite list, that can efficiently be sorted to retrieve its maximal element. In contrast, if *a* is interpreted as a continuous input parameter of the neuronal network *C*, we are faced with $\sup _{a}C(\omega _{1},\ldots ,\omega _{p},s_{t+1}, a)$, where we have to take a supremum over infinitely many values of *a*. This is a non-trivial optimization problem instead of simply sorting a list.

In order to circumvent this problem, we introduce another neural network *A* that maps from the state space to the action-space and may depend on another set of weights $\omega '_{1},\ldots ,\omega '_{r}$. We call *A* the *Actor network*. This is only one possible design choice to solve the issue, but it is the hallmark of so-called *actor-critic methods*. Instead of a neural network one could assume a functional form of *A* that allows for efficient solution of the maximization problem, but that would be more similar to so-called policy gradient methods. The idea of the neural network based approach is that by updating

8 $$\begin{aligned} \sup _{\omega '_{1},\ldots ,\omega '_{r}}\sum _{t} C(\omega _{1},\ldots ,\omega _{p},s_{t},A(\omega '_{1},\ldots ,\omega '_{r},s_{t}) ) \end{aligned}$$

iteratively the actor becomes an approximation of the function mapping states to ideal moves, thus

9 $$\begin{aligned} \sup _{a}C(\omega _{1},\ldots ,\omega _{p},s_{t}, a) \approx C(\omega _{1},\ldots ,\omega _{p},s_{t}, A(s_{t})). \end{aligned}$$

Overall, we simplify (7) to

10 $$\begin{aligned} (1-\alpha ) C(\omega _{1},\ldots ,\omega _{p},s_t,a_t) + \alpha [R(s_t,a_t) +\gamma C(\omega _{1},\ldots ,\omega _{p},s_{t}, A(s_{t}))], \end{aligned}$$

leaving us with the actor-critic update rule. If the actor network is chosen to output a single deterministic value $a_{t}$ for each input state $s_{t}$, we obtain the update rule that is characteristic for a deep deterministic policy gradient (DDPG) learning algorithm.

## Price-Competition in a Symmetric Capacity Constrained Duopoly

In order to test the performance of our algorithms to derive Nash equilibria in a strategic bidding context, we first discuss our benchmark scenario. We deliberately chose a simple framework that has an analytical (static) solution but at the same time captures the nature of price competition in a sealed bid uniform price auction setting. Overall, we aim to asses the performance of a continuous reinforcement learning algorithm (DDPG). Consequently, the benchmark scenario should have a straight forward continuous formulation. Moreover, we do not want to limit our analysis to the relative performance of our algorithm with any particular set of competing algorithms but we desire some form of absolute performance measure. Hence, we require a benchmark scenario that is analytically solvable. We have kept the scenario simple so that all Nash equilibria can be computed by hand. This allows us to comment on every equilibrium and analyze how hard it is to find respectively.

We study the following scenario: Assume two identical players, *i* and *j*, competing against each other in prices. Both players control a capacity ${\overline{q}}_i={\overline{q}}_j={\overline{q}}>0$ allowing them to commit to produce a homogeneous product. Both players have equal marginal cost $c_i=c_j=c \ge 0$. Players participate in a uniform price auction setting by submitting price offers. We write the price offers without loss of generality as $p_i \le p_j \le {\overline{p}}$. Offer prices are constrained by a price cap (${\overline{p}}$). If both players were submitting the same offer prices to the market, each player may sell one-half of the total demand (“tie breaking rule”). We furthermore assume inelastic demand *D*, that we require to be within ${\overline{q}}< D < 2{\overline{q}}$ to ensure non-trivial solutions.

For any given set of parameters (${\overline{q}},c,{\overline{p}},D$) and the tie break rule as described above, we can compute an interval of Nash equilibria. We derive the Nash equilibria in Proposition 1. There, we find and characterize a non-trivial range of equilibria that is well above the marginal costs of the players.

This allows us to contrast numerical results against a well understood background. We have conducted all our numerical simulations with the following set of parameters, ${\overline{q}} = 50, c = 20, {\overline{p}}= 100$, and $D=70$, unless specified otherwise. We emphasize that the condition ${\overline{q}}< D < 2{\overline{q}}$ is crucially needed for the existence of non-trivial Nash equilibria. Alternatively, one may consider either the “uncompetitive” case $2{\overline{q}} \le D$ or the “ultra-competetive” case $D \le {\overline{q}}$.

In the “uncompetitive” case all capacity is sold, regardless of the players behavior. Consequently, all strategies, where at least one player offers at the price cap are within equilibrium. We consider this case to be a trivial case, that we do not discuss further.

In contrast, the “ultra-competetive” case $D \le {\overline{q}}$ essentially renders the capacity constraint irrelevant and thus reduces to standard Bertrand competition, where both players offering their marginal costs constitutes the equilibrium strategy in this case. Although the predictions for the standard model of Bertrand competition are well-known ($p_i^* = p_j^* = c$), this case is not necessarily trivial when the goal is to learn this outcome through a black-box method. We believe that complying with game-theoretical predictions for the special case of (non-capacity) constrained Bertrand competition is an important minimum requirement in order to trust a learning algorithm. Therefore, we include it in our discussion of the results later on.

If we would discretize the players action space, the two player condition allows us to easily visualize the games payoffs as a bimatrix. Moreover, this discretization matches the discrete action space of *Q*-learning. Computing Nash equilibria can be done straightforward from the bimatrix. Each best response can be read of row-wise and column-wise. We find all equilibria precisely, where row- and column-wise best responses match. In contrast, computing the equilibria of the continuous case requires a thorough analysis. Our scenario is chosen precisely such that this effort remains low, however in more general scenarios we have no guarantee of analytical solvability.

### Proposition 1

Consider an uniform price auction with two participants and eligible offers that may be placed in the interval $\left[ -\infty , {\overline{p}} \right]$. If both participants offer a fixed quantity ${\overline{q}}$ of an indistinguishable product, whose production involves a constant per unit production cost of *c* and the demand *D* lies within ${\overline{q}}< D < 2{\overline{q}}$, then there exist w.l.o.g. a number of tuples of equilibrium strategies $p_{L}<p_{H}$. The range of lower equilibrium offer prices being all $p_L$ with

11 $$\begin{aligned} p_{L} \le ({\overline{p}} - c)\frac{(D-{\overline{q}})}{{\overline{q}}} + c, \end{aligned}$$

and the unique higher equilibrium offer price $p_{H}$ being

12 $$\begin{aligned} p_{H}={\overline{p}}, \end{aligned}$$

is the auctions range of Nash equilibria.

### Proof

Let us start by identifying the player’s best responses. We begin by case distinction, either $p_{L}=p_{H}$ or $p_{L} \ne p_{H}$ holds. In case of $p_{i}=p_{j}$ the tie breaking rule applies and each player receives $p_{L} \frac{D}{2}=p_{H} \frac{D}{2}$ Since any player could improve his profit to $p_{H} {\overline{q}}$ by minimally lowering his offer and thus selling its whole capacity, offering $p_{L}=p_{H}$ can not be a best response for any player.

Hence, without loss of generality, we can assume $p_{L}<p_{H}$ (thus allowing us to interpret *L*, *H* as low and high offering respectively). Let us consider the action of the high offering player $p_{H}$. $p_{H}$ is a best response if the high-bidder *H* can neither gain by increasing or decreasing its offer.

Since the high-bidder is the price setting player, we can write the profit of the high-bidder as $\pi _{H} = (p_{H}-c) (D-{\overline{q}}) \le ({\overline{p}}-c)(D-{\overline{q}})$. Thus the high-bidder can always increase its payoff by increasing its offer, unless it offers at the price cap. This makes offering at the price cap the only candidate for a best response of the high-bidder.

It remains to discuss, whether the high-bidder can improve its profit by lowering its offer. Lowering ones offer can only increase ones profit, if one undercuts the competition and thereby gains a large share of the market. This means that in order for $p_{H}={\overline{p}}$ to be a best response the following equation has to hold:

13 $$\begin{aligned} (p_{L}-c) {\overline{q}} \le ({\overline{p}}-c) (D-{\overline{q}}), \end{aligned}$$

otherwise the high-bidder might gain by undercutting the low offering player. For the low-bidder in-turn, all prices below the price cap yield the same profit and offering at the price cap leads to a tie that decreases its profit. Therefore, the low-bidder is too playing a best response. Hence, if Eq. (13) holds both player are playing a best response and thus a Nash equilibrium.

Therefore, the high offering player is completely fixed, however the low playing player has a range of possible equilibrium strategies. To characterize these, we go on to reformulate the above condition to

14 $$\begin{aligned} (p_{L}-c) \le ({\overline{p}}-c)\frac{(D-{\overline{q}})}{{\overline{q}}}. \end{aligned}$$

Now considering that ${\overline{q}},c,D$ and ${\overline{p}}$ are fixed by our assumptions, we have found a closed representation of $p_{L}$ being in equilibrium and the statement to be shown follows. $\square$

We briefly comment on the implications of Proposition 1. First, we see that the interval of equilibria depends on production costs, the ratio of sales and the price cap.

In our case the assumption of completely inelastic constant demand leads to the price cap being the only constraining factor on the offer prices of the high offering players. If demand was elastic the consumers would put an implicit price cap and therefore constrain the suppliers in their ability to determine the market clearing-price. Note that the high offering player needs to be always bounded from above as required in Eq. 13.

## DDPG Learning in Uniform Price Auctions

Deep Deterministic Policy Gradient learning (DDPG) is a successor of classical RL (*Q*-learning) allowing for continuous action and state spaces. Clearly, storing all function evaluations in a *Q*-table becomes infeasible in a continuous setting. Therefore the *Q*-table is only approximated using neural networks. DDPG employs a so-called Actor-Critic design. Hence, actions and states qualities are evaluated by two differing neural networks: the Actor network and the Critic network respectively. For example in the bidding context, the actors submit offer prices and the critics collect the offers and past rewards in order to predict future hypothetical profits. Once all offers are submitted, the market clearing determines winners and losers of the auction and awards revenues to the agents. Agents process this information and update their neuronal networks to incorporate the new information gained by the current auction.

DDPG is a well-tested and successful algorithm, that has performed well in control tasks in a range of continuous environments, [e.g., several robot motion control tasks (Zhang et al. 2021), autonomous driving (Yi 2018)]. In this section, we proceed to demonstrate that DDPG can also learn strategic behavior in continuous environments such as uniform price auctions. Moreover, we give a detailed presentation of our DDPG implementation and discuss the impact of relevant design choices such as learning rate, memory and normalization methods.

Run-time ends are usually not determined by a convergence criterion but by fixed iteration numbers (i.e., 100,000 or 20,000). However, we employ convergence criteria for the evaluation of statistics. Convergence is always understood with respect to the underlying game-theoretic Nash equilibrium. The Nash equilibrium is provided by Proposition 1 for the capacity constrained case and by the 2nd lowest production cost in the unconstrained case. We average the last 5000 actions and compare their deviation from the game-theoretic prediction. Simulations with averages that lie within a tolerance of 2 standard deviations of the minimal noise are considered converged. Note, that we employ an adaptive noise with larger standard deviation in the beginning and a lower standard deviation towards the end of the run-time.

We deploy DDPG to explore its efficacy to find equilibria in a competitive bidding environment. There is not yet a standardized protocol that assesses these complex algorithms and one of our contributions is a first framework to perform such an analysis. Furthermore, we regard DDPG as a particularly successful instance of the much larger family of deep learning algorithms. We believe that our results can be taken as exemplary for treating the much broader question of *deep learning’s efficacy for simulations of strategic bidding behavior*.

In order to test DDPG, we rely on the simple but non-trivial benchmark example described in Sect. 4, with an analytical solution. This is important because it allows us to classify whether we successfully learned an equilibrium outcome. We interpret convergence towards an equilibrium outcome as a sign of successful learning. Finally, we perform statistics on the strategies the algorithm converged to after a fixed learning period. The basic benchmark scenario is kept constant in order to evaluate the influence of several parametrizations of the algorithm.

One of the main challenges of assessing reinforcement learning algorithms in general is their dependence on a number of user-defined “hyper-parameters.” Already for simple *Q*-learning, parameters such as learning rate, discount rate, and exploration-exploitation parameters will affect the algorithm’s speed and convergence. When approximating the *Q*-function by a neural network, additional parameters, such as the memory buffer size, need to be chosen. The DDPG framework consists of two neural networks, hence, all the aforementioned parameters will affect solution quality and solution performance but the benefit of this approach is that it is able to treat continuous state-action spaces.

There is no generally agreed prescription on how to select hyper-parameters, however, specific problem domains have distilled their experience into broad design guidelines for selecting these parameters. For instance, image recognition has nowadays been associated with convolutional neural network architectures (CNN’s) and image generation is typically relying on generative adverserial networks (GAN’s). Employing this kind of methods to market equilibrium problems is relatively new and therefore we can not draw on any such prescriptions. Our design philosophy is to closely mimic the parametrization of the seminal paper that introduced continuous actions-space deep learning (Lillicrap et al., 2015).

This design was chosen because it has demonstrated versatility and efficiency in continuous control problems. We deploy the concept provided by Lillicrap et al. (2015) because an established deep reinforcement learning design for decision making in economics has yet to be developed. The problems in Lillicrap et al. (2015) usually are controlling for a small number of parameters with different but continuous action space ranges (i.e., bearing, throttle, brake). This setting is similar to our auction market problem where market participants can choose price, quantity, or both.

**Fig. 1:**
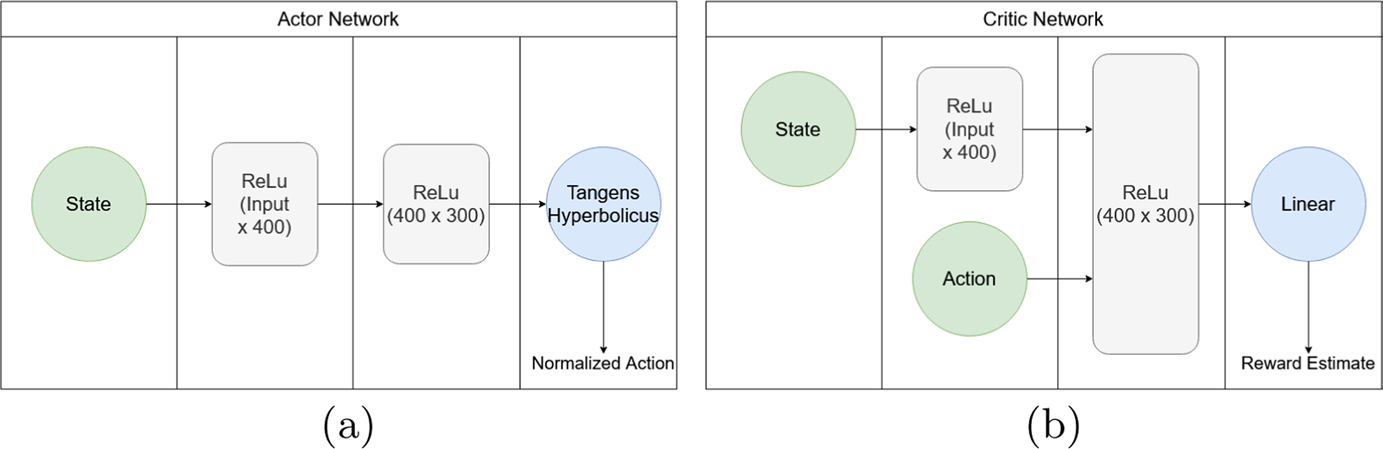
Schematic representation of neural network architecture. *Notes:* The actor network (Panel **a**) uses one layer to process state information, subsequently performs one coarse graining step and uses a tangens hyperbolicus layer to normalize outputs between $[-1, 1]$. Critic architecture (Panel **b**) is similar, although action input is directly fed into layer 2. The output layer is linear. All hidden layers use rectified linear activation units functions

It is worthwhile to reexamine the network design of Actor and Critic depicted in Fig. 1. Here it is apparent that the Actor is slightly simpler in design as opposed to the asymmetric design of the Critic. The Lillicrap et al. (2015) design recognizes that the Critic processes more complex inputs (both states and actions) than the Actor (only states). This difference is mirrored by the inputs of the Actor taking two different paths through the network. Indeed, the processing of state space information traverses identical layers in the Actor and the upper branch of the Critic alike. In contrast, the input actions traverse a smaller rectified linear units (ReLU) layer. This design implicitly assumes that it is easier to encode the impact of one’s actions (as we do have control over them) than the impact of environmental influences (where a large number of confounders may be present). We believe that this economical approach in representing actions is advantageous if the agent controls a small number of distinct actions such as a single-unit price offer.

We precisely list all relevant hyper-parameters that are related to deep learning in Sect. 5.3. In Sect. 5.5, we systematically vary the most relevant hyper-parameters and report which changes have been beneficial to derive equilibrium outcomes in bidding problems. It is our hope to contribute thereby to building up similar domain specific recommendations for deep learning in energy systems analysis as are currently available for image recognition.

### Main Benchmark Scenario: Capacity Constrained Bertrand Competition

We briefly recapitulate the structure of our benchmark scenario detailed in Sect. 4. Our setting consists of a duopoly competing in offer prices. The competition takes place through an uniform price auction mechanism. The auction mechanism has the objective to maximize welfare given the firm’s offer prices and returns a single (uniform) market clearing price and market clearing quantities for both players. We assume that both players have a fixed capacity and submit offer prices simultaneously to the auction mechanism. The static game has a continuum of equilibria that can be described analytically. In the learning context however, the one-shot game will be iterated many times and agents may learn how to optimally navigate in such a setting.

We assume symmetry, i.e., both players are identical in terms of cost and maximum capacity of 50 they control. Both players are assumed to always offer their full capacity and may submit offer prices in a continuous interval that we have normalized between $[-100,100]$. We assume a production cost per unit of 20 and constant inelastic demand of 70.^8^

We parameterized our benchmark model such that demand is strictly larger than each firm’s capacity. Hence, both firms will have positive market clearing quantities. However, due to the uniform pricing auction setting, the marginal firm, i.e., the firm that will set the price, will produce less than the infra-marginal firm. Hence, the trade-off in this game is between setting the price but selling less, and being infra-marginal (not setting the price) but selling full capacity. Clearly, the latter strategy will dominate in terms of per-unit profit given one player is setting a high price and that both players are symmetric. We find that equilibrium strategies typically converge to states where one player is offering at the price cap and the other one far below—a behavior consistent with the analytical equilibrium description.

In the completely symmetric scenario, which agent ends up as high- or low bidder is a the result of a complex interaction of network initialization, the random seeds, initial exploration and the learning rate. On average both players end up 50% as high-bidder and 50% as low-bidder. However, this dependence on randomness stems essentially from the strong symmetries of the benchmark scenario. In the main benchmark scenario, we mostly refrained from “favoring” a player, except in the asymmetric production cost scenario, where the low-bidding player is determined by the more favourable cost structure.

**Fig. 2:**
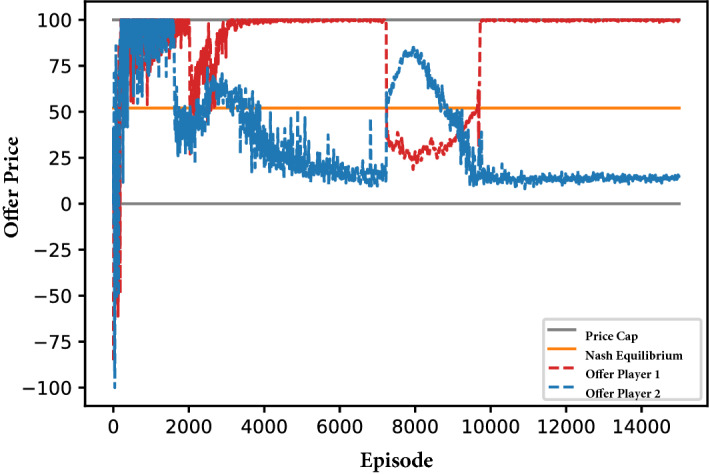
Exemplary learning progression. *Notes:* The figure shows the development of offer prices in a duopoly. Each point corresponds to a players offer price in a given episode. The figure shows different phases of the learning progress throughout an exemplary DDPG run. We can distinguish: **a** Episode 0–2000: random exploration, exploration of action space limits; **b** episode 2000–4000: competition and exploration of opponents reactions; **c** episode 4000–7000: initial relaxation to equilibrium with high variance; **d** episode 7000–9000: attempted exploration of alternative strategies; **e** Episode over 9000: final stabilization to equilibrium with low variance

The advantage of deploying DDPG to the problem rather than *Q*-learning is that the former is able to handle continuous action spaces rather than only discrete actions spaces. Nonetheless, with the right parameterization, we achieve a reliable convergence of the DDPG algorithm despite the continuous action space. For instance, in Fig. 2 we depict the learning progression of a well-tuned DDPG run.

**Fig. 3:**
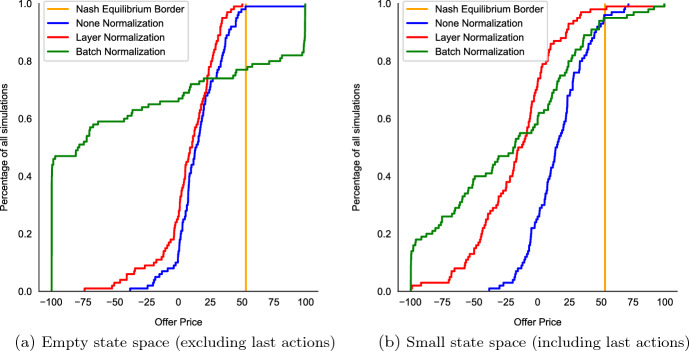
Distribution of Offer-heights depending on Normalization. *Notes:* We compare 100 unnormalized (blue), batch normalized (green), and layer normalized (red) DDPG runs in the benchmark scenario described in Sect. 4. Panel **a** depicts runs with empty state space and Panel **b** depicts runs with explicit state space information about last round actions. We plot cumulative densities with respect to offer height, thus showcasing the distribution of the low-offering player offers. Offers left of the yellow bar correspond to an equilibrium strategy. The graphs plot the percentages of all offers that attained at least the indicated height. Note that the offer price was re-scaled to its actual range after normalization

DDPG also remains effective in finding equilibrium outcomes. Figure 3a, b depict the share of 100 test runs that have terminated in a Nash equilibrium state. The blue, green, and red line describe runs with differing normalization schemes (see Sect. 5.4.2), the height of these lines intersection with the orange Nash equilibria border is the ratio of runs that converged to equilibrium within 15,000 episodes.^9^ There, we see that the best performing method (i.e., layer normalization, red line) achieves convergence rates of $100\%$ and $99\%$ in the small and large state space case respectively. Therefore, it is evident from Fig. 3a, b that it is possible to attain reliable convergence in continuous state-action spaces with DDPG.

### Alternative Benchmark Scenario: Unconstrained Bertrand Competition

The ratio between demand and the capacities of the agents is a critical parameter. We have discussed the details in Sect. 4, where we argued that the most interesting case is ${\overline{q}}<D<2{\overline{q}}$, as covered in our standard benchmark case. Nonetheless, $0 < D\le {\overline{q}}$ is worth a brief discussion, since it reduces the problem to an unconstrained Bertrand duopoly. It is well-known that the only equilibrium in this case is the one, where both players offer their marginal costs. Albeit, being of minor theoretical relevance, reproducing the behavior of Bertrand competition constitutes an important first validation of our algorithms agreement with established theory. Indeed, some computational methods turned out to have problems reproducing the theoretic predictions of Bertrand competition. For instance, Noe et al. (2012) report that genetic algorithms tend to not converge to Bertrand equilibria in a single unit setting, regardless of 1st or 2nd price auction designs and both with fixed and uncertain valuations. In contrast, DDPG turns out to reproduce theoretical predictions.

In a slight departure from our standard benchmark case, we have performed a couple of algorithm runs implementing a standard Bertrand duopoly with ${\overline{q}}_1={\overline{q}}_2=D=50$ instead of our standard choice ${\overline{q}}_1={\overline{q}}_2=50, D=70$.

**Fig. 4:**
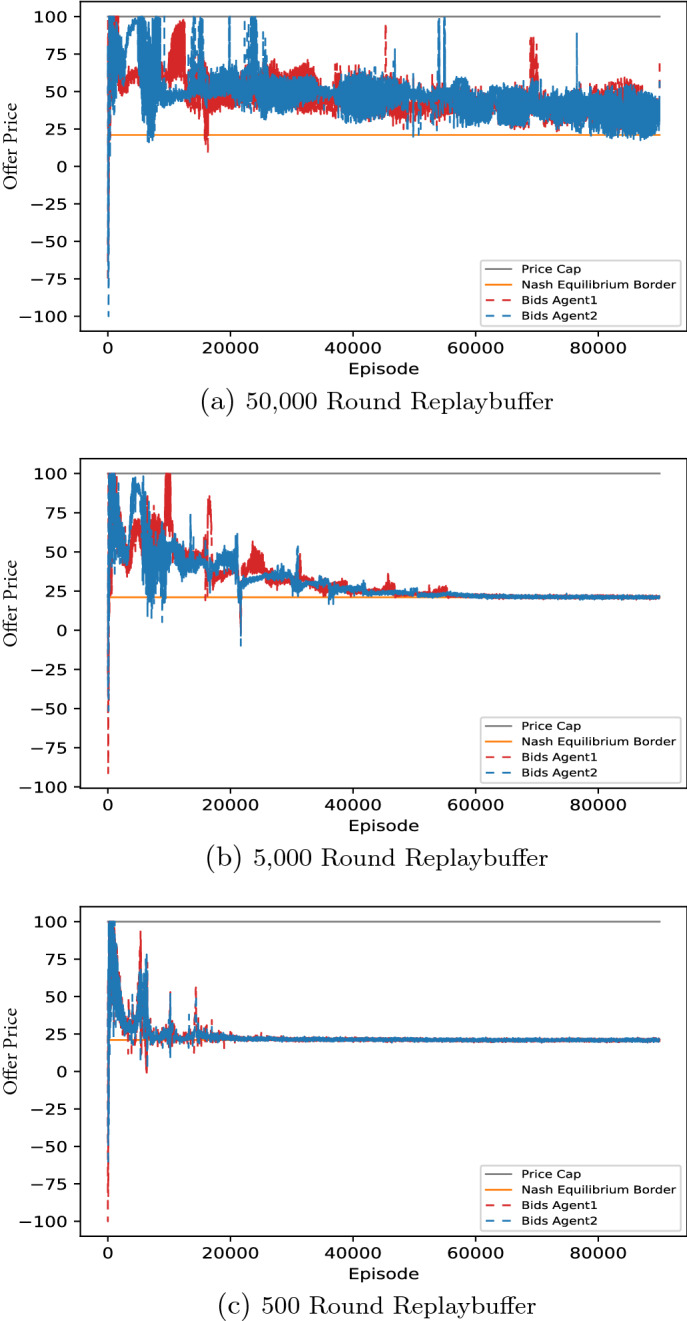
DDPG convergence times of unconstrained bertrand games depending on replaybuffer size. *Notes:* Figures show the influence of replaybuffer size on convergence in an “ultra-competitive” scenario, where $D \le {\overline{q}}$. Each panel depicts the fastest converging DDPG run out of 10 identical runs. Here, the convergence times are from top to bottom **a** above 100,000 episodes, **b** around 50,000 Episodes, and **c** around 20,000 episodes, hence convergence times are *decreasing with smaller replay buffer sizes*

In essence, we find that DDPG converges towards marginal costs in the standard Bertrand duopoly as expected. These findings remain robust under differing cost-structures and player numbers. In Fig. 4, Panel (a)–(c), we have chosen a duopoly with symmetric marginal costs , purely due to reasons of convention (i.e., $c_1=c_2=20$). This represents the standard situation, when studying Bertrand competition. DDPG converges completely analogously with different symmetric cost levels and asymmetric costs. For instance, asymmetric costs scenarios (we simulated the case $c_1=0$ and $c_2=20$) converges reliably too.^10^ That being said, DDPG converges to the higher of the two production costs, which generalizes the case of symmetric costs consistently.

More generally, for oligopolies with more than two firms the above still holds. As long as every company can satisfy the demand on its own, DDPG converges towards the 2nd lowest marginal costs. In other words, the firm with the 2nd lowest marginal cost will price-constrain the firm with the lowest marginal cost. Hence, the cost structure does not affect convergence and multi-player behavior is consistent with duopoly behavior. We want to remark that Bertrand competition with asymmetric cost structure is a prime example where continuous algorithms such as DDPG outperform discrete algorithms such as *Q*-learning. For instance, lets call the smallest difference between two discrete actions the action step-size, $$\epsilon _{d}$$. The action step-size effectively introduces an error of up to $$\epsilon _{d}$$ if $$c_2$$ does not directly intersect with the discretized offers. For large $$\epsilon _{d}$$ this is of relevance, since we know that the number of states to explore goes to infinity as $$\epsilon _{d} \rightarrow 0$$. Hence, discrete algorithms exhibit a clear precision run-time trade-off in this case. For instance, the largest action step-size found in our literature discussion of *Q*-learning reaches up to $10/MWh (see Table 1 or Table A.11, Appendix A, in Aliabadi et al., 2017).

However, there exists a caveat: the speed of convergence. Indeed, it is sometimes possible that resulting offer prices approach the marginal costs at a speed leading to convergence times of roughly 100,000 episodes. Well parametrized runs of capacity constrained Bertrand duopolies typically do not exceed convergence times of 20,000 episodes.

Moreover, we were able to identify at least two criteria that control the convergence times of unconstrained duopolies: the size of the memory buffer and the noise decay rate. Indeed, this case makes it particularly apparent that the size of the memory buffer needs to be chosen carefully. Both too large and too small buffer sizes negatively impact the convergence of the “easy” unconstrained problem. Similarly, the noise decay needs to be adjusted, this finding is however expected (well-known as exploration-exploitation trade-off) and similar in all RL algorithms. We discuss the details in Sect. 5.6.

We want to remark that (even extremely) slow convergence to an equilibrium (such as marginal costs in unconstrained Bertrand competition) by no means contradicts any fundamental game-theoretical predictions because GT does not predict *how* or *how fast* an equilibrium will be found. GT solely predicts that eventually players ought to arrive and remain within equilibrium. It may well be the case that certain equilibria are relatively slow to learn despite having well-known theoretical justifications.

### Tuning Parameters

DDPG is considered a so-called actor-critic method, thus it uses two neural networks (the actor and the critic network) in parallel with possibly differing design choices. Hence, DDPG requires a large set of parameters to be tuned. A relevant contribution of our work is to evaluate sensible values and relevant parameters for the problem domain of uniform price auctions. However, due to the many possible interactions between parameters a selection has to be made, which parameters are investigated in detail and which are held constant. We have deemed the following parameters to be most relevant and thus studied their significance in Sect. 5.5 through a variational analysis, in particular, the *actor learning rate*, the *critics learning rate*, inclusion of *last rounds actions in the state space*, and several *action normalization schemes*.

While we held *replay buffer size* fixed in the main benchmark scenario (capacity constrained Bertrand competition), we performed a variational analysis of replay buffer size for the unconstrained Bertrand duopoly. Moreover, we have found significant differences between constrained and unconstrained Bertrand duopolies. Indeed, in the unconstrained case we have found the *replay buffer size* to be the decisive parameter alongside the *noise decay rate*, although these have not been relevant for convergence in the constrained case.

Apart from these variational analyses, the following parameters, chosen almost entirely in accordance with Lillicrap et al. (2015), have been held fixed: the choice of an almost empty *state space* except for the inclusion or exclusion of last rounds actions, the *optimizer*, *actor network design* including depth and hidden layer, *critic network design* including depth and hidden layer, *soft-update rate*, $\tau$, *noise*, and the use and size of a *replay buffer*. We have summarized our choice of parameters in Table 2 and depicted the overall structure of our neural network architecture and the flow of reward-action-signals in Fig. 5.

**Table 2:** Model and hyper parameters

Model environment parameters		General hyper parameters	
Capacities (${\overline{q}}$)	50	Soft-update rate ($\tau$)	0.001
Marginal costs (*c*)	20	Discount rate ($\gamma$)	0.99
Price cap (${\overline{p}}$)	100	Max memory size	50,000
Demand (*D*)	70	Batch size	128
		Total runs	100
		Episodes per run	15,000

Noise hyper parameters		Neural network hyper parameters	
Mean ($\mu$)	0	Learning rate actor	0.0001
Variance ($\sigma$)	0.1	Learning rate critic	0.001
Regulation coefficient	10		
Decay rate	0.001

**Fig . 5:**
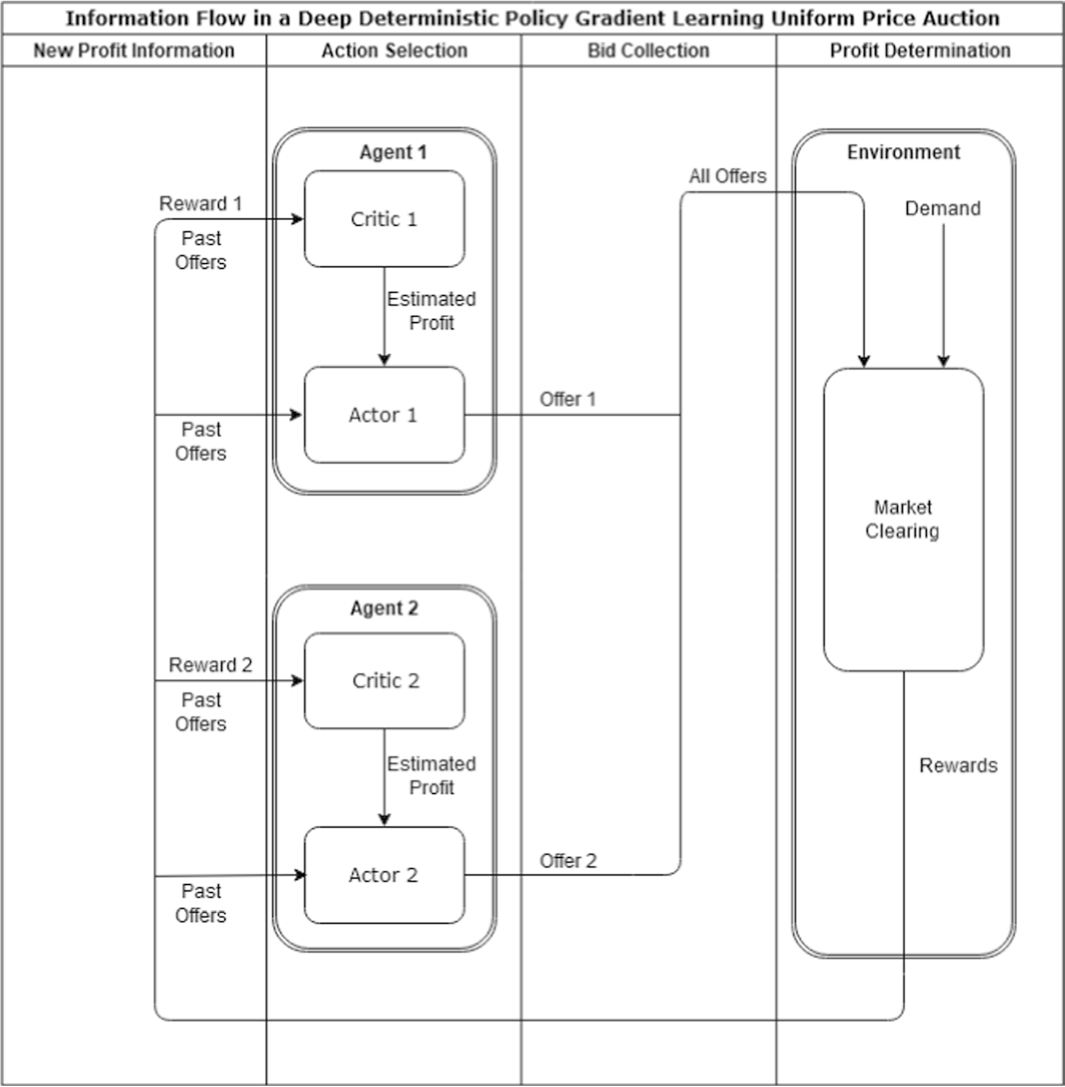
Depiction of the employed multi-agent neural network architecture. *Notes:* We depict the interaction between agents and environment in our uniform price auction scenario. Each agent is equipped with two neural networks: the critic and the actor. In each round the critic processes the actual rewards and produces future reward estimates, that are in turn fed to the actor who uses them to determine their offer prices. In case of explicit state space memory, all neural networks receive last rounds offers as state space information. All agents submit their offers to the environment. The environment combines all submitted offers with demand information and a market clearing subroutine. The market clearing determines the market price, sold quantities and revenue. Revenue is turned into profit within the environment by subtracting costs. Here, profit translates to the reinforcement learning notion of reward. Finally, rewards and last turn offers are relayed back to the agents, who use it to determine next rounds offers. This completes a single learning cycle

#### State Space

The most fundamental modelling choice in any RL algorithm is how the state-action space is modelled. In our setting the action space is the set of possible offer prices that can be submitted to the market clearing algorithm. In terms of the state space it is less clear what should be encoded in order to capture the dynamics of an environment. We believe that including richer state spaces is a very promising line of future research, but have decided to keep state information limited to keep the analysis tractable. Consequently, the state was chosen to be almost empty with one exception: the offer prices submitted to the market clearing of the previous period. We emphasize that all environment parameters (i.e., capacity, marginal costs, price cap, and demand) have been kept constant, but have not been included explicitly within the state space. Hence, all information regarding the environment parameters is learnt instantly during the algorithm without input from our side. Although DDPG includes a parallel memory mechanism (the replay buffer), we opted to explicitly include this information in the state space. Indeed, we find that such an explicit representation of memory impacts the resulting behavior.

#### Optimization Routine

Deep learning algorithms rely on solving numerous successive optimization problems when updating neural networks, hence the choice of solver is relevant for the algorithms behavior.

We have employed the ADAM solver (Kingma & Ba, 2015). ADAM is not only used in Lillicrap et al. (2015) but can currently be considered as the state-of-the-art implementation of stochastic gradient descent that is employed throughout the deep learning community. ADAM’s popularity comes from the fact that it is an adaptive solver which adjusts initial learning rates and hence promises to work robustly within a range of learning rates. Nonetheless, we find significant influence of learning rates on the convergence of the algorithm.

Moreover, we comment on the common observation that ADAM’s performance is not scale independent. In our opinion this is an undesirable side-effect. However, this effect seems not be limited to the ADAM solver but is consistently reported throughout the deep learning community (van Hasselt et al. 2016). This leads to the counterintuitive fact that problem hardness is affected by the absolute values of the encountered rewards and actions. Nonetheless, we acknowledge ADAM’s prevalence in the deep learning community. Most deep learning practitioners therefore employ a down-scaling of the relevant parameters prior to learning and a subsequent re-scaling after learning to match the environments scaling again. We follow this common practice and find considerable impact of re-scaling in general and the employed scaling method in specific.

#### Actor and Critic Network Design

The neural network of the *Actor* consists of four layers, where the first layer is the input layer corresponding to the size of the state, and the second and third are rectified linear units (ReLU) layers. The hidden layer has a size of 400 input nodes and 300 output nodes. An actor network needs to output an action. The final tangens hyperbolicus layer reduces the output to one node, since each agent can only place one offer per episode. The choice of the tangens hyperbolicus activation function may seem exotic at first glance, however it is motivated by mapping any desired interval to the $[-1,1]$ interval. This re-normalizes the action space to a value range, where the ADAM (Kingma and Ba 2015) optimizer operates reliably. Therefore, the actor network’s output is a normalized action and needs to be re-normalized to make sense within the context of the environment.

The *Critic* neural network consists of three layers, where the size of the first layer is the size of the *state*, the second ReLU layer is also 400 plus the size of the *action* vector, the third layer has also a size of 300 and the last layer has the size of the *action* vector, since a *Q*-value for every value within the *action* vector is needed. Therefore, the critic has a certain asymmetry in design. State information traverses a one layer deeper network than action information. Including the *action* only from the second layer is suggested by the authors of the DDPG algorithm (see Lillicrap et al., 2015).

All neural network weights are initialized by PyTorch standard initialization. The employed initialization differs slightly between layer types. Tanh layers are by default initialized by Xavier initialization, while ReLU layers use Kaiming initialization.

#### Noise

Noise is an essential part of any RL algorithm as it enforces exploration. In principle, the output of a neuronal network is always deterministic. For instance, an Actor neuronal network will always recommend the same action (in our case the offer price) given the same state space. To ensure that the algorithm also explores alternative strategies, noise is introduced. Noise is a normal distributed variable with mean zero and parametrized variance that is added to the actor recommended action. Typically, the variance of the noise is set to a high initial level which decays^11^ over time until a minimal noise variance level is reached.

Lillicrap et al. (2015) originally used an Ohrensteil-Uhlenbeck noise, but recent research showed that a normally distributed Gaussian noise performs just as well (Fujimoto et al., 2018). Hence, we have opted for Gaussian noise, since it can be run with fewer hyper-parameters thus facilitating isolation of relevant parameters.

We decided to use normally distributed Gaussian noise with mean $\mu = 0$ and variance $\sigma = 0.1$. Additionally we apply a regulation coefficient to move the mean, to enlarge the starting noise to further ensure that the whole *action* space is explored adequately. To make sure that the algorithm converges with time, there is also a decay rate applied to the noise. The decay ensures that the noise gets smaller with each episode, until a defined minimum noise setting is reached.

#### Replay Buffer

Another feature of DDPG is the replay buffer. A replay buffer stores a (typically large) number of past rounds. In our case up to 50,000 rounds are stored in the memory buffer. Initially, the buffer is empty and every action is stored within the buffer. The memory buffer is managed according to the first-in, first-out principle i.e., once 50,000 memorized actions are stored, every new action is saved in the buffer, at the expense of deleting the oldest memorized action to adhere to the memory limit.

In the standard benchmark scenario the replay buffer was a fixed parameter with size 50,000. In this case the buffer is chosen so large, that memory constraints should not influence the algorithms outcome. It is possible that the large buffer size may have slowed convergence, but due to us being able to attain convergence with proper learning rate choices, we opted not to vary the buffer size.

Neural networks do not contain an exact description of past rounds, but are adapted iteratively by minimizing their predictions deviation from a set of target states in each update that learns new incoming information. This allows neural networks to remain small, when scaling up the size of the state-action space, with only minor prediction losses. However, neural networks of DDPG should incorporate both new information, but also be able to retain past knowledge. For this reason, every update of a neural network randomly draws a number of old rounds from the memory buffer. The replay buffer aims to statistically represent the past. Then the network is adapted to attain minimal loss on the new rounds and the rounds drawn from the memory buffer.

The replay buffer is yet another feature of deep reinforcement learning methods. In contrast, classical reinforcement learning such as *Q*-learning usually has no directly corresponding type of memory. In *Q*-learning, memory can also be included into the state space. However, the replay buffer is in fact a second type of memory, alongside a possible inclusion of for instance last round actions into the state space. Experiments lacking an explicit memory representation in terms of state space cannot be said to be truly memory-less. Informally, the replay buffer may be seen as a type of approximate “long-term” memory, while the state space memory may be closer to an exact “short-term” memory. An in-depth analysis of the exact interrelation between both memory processes is left to future research.

### Variational Analysis of Learning Parameters

This section covers the impact of parametrization on DDPG’s performance. We will see that correct parametrization is vital to the performance of DDPG, hence we believe that recommendations on best practices in domain specific parameter choices will be a significant contribution. In order to make an informed parameter choice, we vary the following parameters: *learning rate actor, learning rate critic, normalization methods*, and *state space memory*. We analyze the the variations effect on the frequency of found Nash equilibria, which is our main figure of merit. Overall, we vary the two learning rates against each other and the normalization methods against each other. All these are once performed with and without explicit state space memory.

#### Learning Rates

**Fig. 6:**
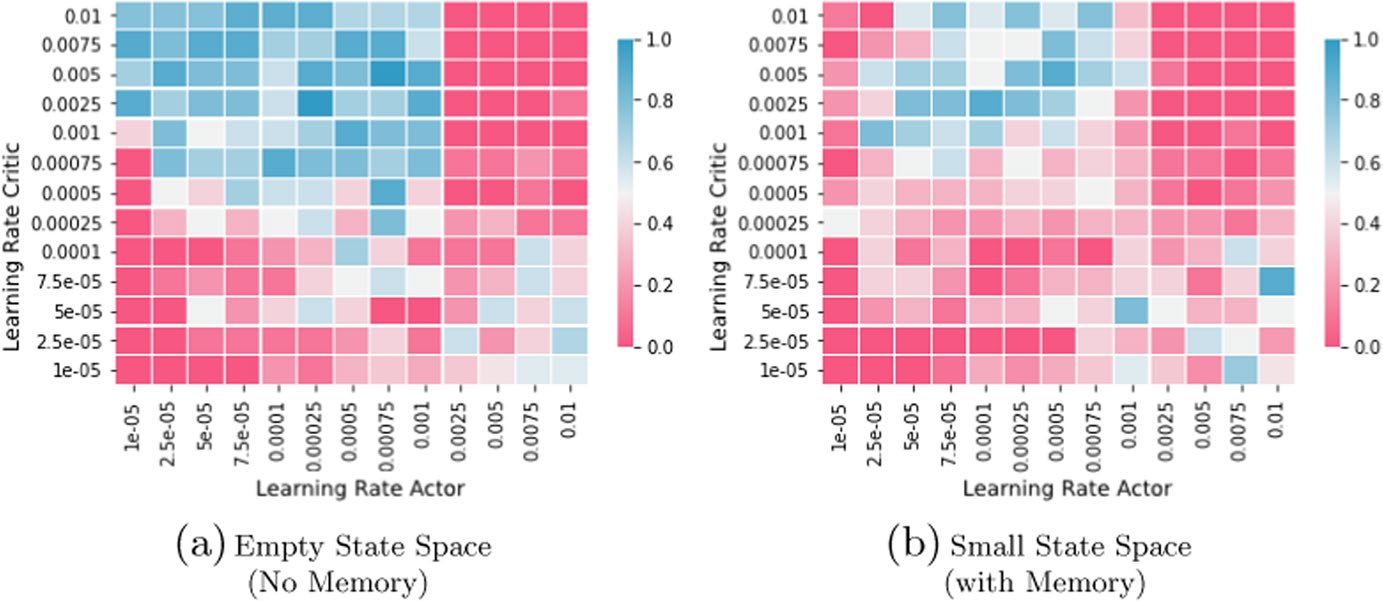
Rate of convergence to Nash equilibrium with varying learning rates. *Notes:* Plots of convergence rate of 10 DDPG runs per pixel depending on actor and critic learning rate. Panel **a** shows an empty state space, while panel **b** is otherwise identical but contains last rounds actions explicitly in the state space. Blue corresponds to high and red to low convergence rates. Panel **a** Shows overall more blue pixels than panel **b** indicating that more complex state spaces are more sensitive to a correct choice of learning rates. Nonetheless, both Panel (**a**) and panel (**b**) contain pixels with 100% convergence rates, implying that with correct parameter choices, complete convergence is possible even with the larger state space of panel (**b**)

A priori it is not clear how to set the learning rates in both of DDPG’s neural networks nor whether they should be the same. Therefore, we vary the actor and critic learning rates independently and around the recommended values in Lillicrap et al. (2015). More specifically, we vary both learning rates in the interval $[1e^{-2},1e^{-5}]$ with four equidistantly chosen learning rate steps per order of magnitude. Consequently, each interval is traversed in a resolution of 13 settings. This means that we have explored 169 learning rate combinations. Each learning rate setting has been run 10 times independently with identical parameter settings. We have performed statistics on the number of runs successfully converging to equilibrium strategies after 15,000 iterated episodes and summarized the outcomes in Fig. 6.

The heatmaps in Fig. 6 show the respective learning rate of the actor and critic networks against the percentage of found equilibrium strategies. Color code blue [red] corresponds to high [low] shares of successful conversions to equilibrium strategies. The two heatmaps in Fig. 6 depict the differences between a memory-less empty state space (Panel a) and a small state space that solely stores the actions of the last round (Panel b).

Generally speaking, we find that rates of high convergence are concentrated in the upper left quadrant of the heatmap. This quadrant corresponds to actor learning rates below 0.0025 and critic learning rates above 0.0025. Choosing learning rates of the actor and the critic to be either very small or very large impairs convergence. Furthermore, the diagonal representing balanced choices does yield sub-optimal convergence results with best results on the $0.001 \times 0.001$ field. While large values of actor learning rates above 0.0025 appear consistently detrimental, with a surprisingly sharp border between 0.001 and 0.0025, critic learning rates may be chosen more tolerantly.

The general pattern is similar in both heatmaps, however it is evident that the blue region of convergence is significantly smaller in the larger state space case in Fig. 6, Panel (b). This shows a visible impact on convergence despite a relatively modest growth in absolute state space size of two extra-variables. Furthermore, it demonstrates that in our specific context, supplying additional information is not necessarily beneficial. Although the smaller region of convergence is a drawback, we point out that this does not imply that the algorithm converges to a lesser degree with more state-information. In fact, this is more indicative of the algorithms sensitivity to parametrization. Both panels in Fig. 6 reach maximal convergence rates well above 90%. This means that in both panels there exist parameter choices that lead to almost guaranteed convergence. However, the number of learning rate combinations that lead to convergence above 90% is lower in Panel (a) than Panel (b). This in turn means, that there are less parameter combinations that lead to reliable convergence in Panel (b), i.e., the case with state space memory. Hence, well-calibrated algorithms converge reliably regardless of state space size, while ill-calibrated algorithms fail to converge. What differs is the number of learning-rate combinations that reach high-convergence rate. Large state spaces work well with fewer learning-rate combinations, thus they are more sensitive to the choice of learning parameters. Therefore, increasing size of the blue areas in the Fig. 6 can be interpreted as a measure of increasing robustness. This indicates a relation between state space size and learning parameter robustness.

**Fig. 7:**
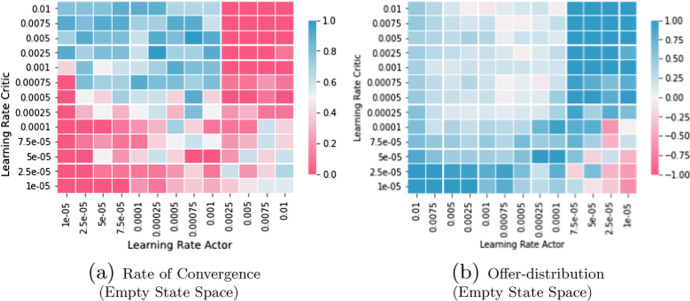
Relating rate of convergence to winning offer-distribution. *Notes:* Panel **a** plots the convergence rate of 10 DDPG runs per pixel depending on actor and critic learning rate. Panel **b** plots instead the average of the 10 runs lower offers. Blue corresponds to high and red to low convergence rates. We can see a similar curved shape in both panels. Typically, low convergence rates correspond to both players offering near the price cap. In contrast, while the top-left and bottom-right corner are both convergent in Panel (**a**), the offer distribution differs significantly in Panel (**b**). All runs were performed in benchmark scenario 4

We have discussed the upper left quadrant in Fig. 4 at length. However, there remains the lower right corner, where we too see convergence in some settings. This corner is starring very low actor learning rates such as 0.01 combined with very low critic learning rates in the order of $10^{-5}$ yielding modest convergence rates. Furthermore, we find that most of these runs are situated on the extreme edges of the action spaces. This is illustrated in Fig. 7, where we contrast the rate of convergence with small state space against the offer-distribution of the low-offering player. We only depict the offer prices of the lower offering player because in equilibrium the high-offering player is supposed to offer at the price cap. Therefore, in equilibrium only the low-offering player has freedom to vary his offer prices. In the lower right corner of Fig. 7, Panel (a) and (b), we see the following pattern: the blue-colored area of converged runs in Panel (a) mirrors the red pattern of extremely low offers in Panel (b). Hence, large actor learning rates combined with small critic learning rates effectively lead to a form of equilibrium selection that favours extremely low offers. It is not entirely clear whether this is desirable convergence in the lower right quadrant results from true learning of the equilibrium. It is possible that these choices are related to “being stuck” at the lower action spaces border. Therefore, we regard the convergence in the lower right quadrant as a less desirable outcome than the convergence in the upper left corner.

To summarize, we find setting the critic learning rate larger than the actor learning rate is beneficial in our setting. While there is a significant range of valid actor learning rates, typically large learning rates are beneficial for the speed of convergence, if convergence is attained at all. Staying within the $10^{-3}$–$10^{-4}$ range delivered the best results. We also point out that the inclusion of state variables is a delicate choice. Our results show that it is possible to attain competitive convergence rates with more complex states. However, the relevance of a correct choice of parameters increases too. Relevant state parameters should be included together with careful inspection of the learning parameters, but we believe that less-informative or even redundant state variables would have a detrimental effect on the algorithm’s performance.

#### Normalization Schemes

One of the methodological differences between classic reinforcement learning (*Q*-learning) and deep learning is the strong prevalence of normalization methods within the latter.

Most machine learning practitioners report significant scale dependencies on the magnitude of the input parameters, when applying deep learning techniques (van Hasselt et al., 2016). This surprising effect means that it may be more effective to learn from inputs in an interval [0, 1] than [0, 1000]. These scale dependencies are observed consistently throughout differing problem domains (Hinton et al., 2012; Krizhevsky et al., 2012), even apparently scale-invariant problem domains. Therefore, down scaling of input data prior to learning and re-scaling to the real problem domains scale after learning is standard practice within the deep learning community.

The potential dependence of model outcomes on normalization distinguishes deep learning from classical methods that is why we provide an in-depth analysis of the impact of normalization on learning. We contrast *unnormalized data* with the two most relevant normalization schemes: *batch normalization* (Ioffe & Szegedy, 2015) and *layer normalization* (Ba et al., 2016). Note, that within DDPG in each step of learning, several neural networks are adapted to a batch of several generated observations. Each individual observation is represented by a vector, where each component represents a feature of the state-action space. Batch normalization curbs the variance between several input vectors (i.e., samples) and layer normalization reduces the variance between the components (i.e., features) of an individual input vector. This means that batch normalization normalizes separately each individual feature’s range over several observations. In contrast, layer normalization normalizes the features within a single observation. All three normalization approaches are once applied to a problem formulation without state variables (i.e., only actions as inputs) and a formulation that includes last rounds action as state-variables (i.e., “single round memory”) as elaborated in Sect. 5.3.1.

Figure 3a, b contrast the impact of different types of normalization schemes and state space memory information on the number of learnt equilibrium strategies for the low-offering player. The figures of merit depict the percentage of offers within equilibrium. In order to collect statistics, all normalization schemes have been iterated 100 times. Remember that equilibrium outcomes in our setting are characterized as one player offering at the price cap and its opponent offers below the threshold given in (11). The threshold is determined by the parameters of the game, i.e., price cap, marginal costs, and the ratio of capacity to residual demand. For our standard model parameters (see Table 2), this threshold is calculated as $({\overline{p}}-c)\frac{(D-{\overline{q}})}{{\overline{q}}} + c = (100 - 20)\frac{70-50}{50} + 20 = 52$. We depicted the threshold as the yellow vertical line in Fig. 3a, b. The three remaining lines in each figure represent the cumulative density function, i.e., the distribution of offers derived by differing normalization schemes ordered by their offer height. The intersection point between one of these lines and threshold (yellow) is key, because its height on the y-axis gives the percentage of equilibrium compatible offers. Here, the higher the point of intersection on the y-axis, the better.

We find that in the memory-less case (Panel a) with small state space, layer normalization (red, approximately 100%) outperforms “no normalization” (blue, approximately 99%) as well as batch normalization (green, approximately 77%). In Panel (b), we conduct the analysis, explicitly including the last round of actions into the state space. As before, layer normalization (red, approximately $98\%$), outperforms the unnormalized scheme (blue, approximately 93%) and batch normalization (green, approximately 92%). Layer normalization has consistently performed best in our setting and is thus recommendable with the caveat that even unnormalized runs performed relatively well. Batch normalization performed worst. However, we find the strong sensitivity of batch normalization to the increased state space size remarkable. We reemphasize that the increase in size of two additional state variables is relatively small compared to state spaces size common in e.g. the ATARI domain (Mnih et al., 2015). Nonetheless, batch normalization improved its performance by at least 20%, while the other methods got slightly less efficient. This motivates us to conjecture, that batch normalization might proof robust when scaling-up the state space complexity. Moreover, the relatively good performance of unnormalized runs might be lost in larger state space, since even a small increase notably had a relevant effect on the unnormalized runs.

Aside from the impacts of normalization on rates of equilibrium convergence, we observe remarkable qualitative differences in the arising offer distributions. For instance, batch normalization exhibits a much flatter offer distribution compared to the unnormalized case at almost identical rates of convergence in the large state space case (see Fig. 3b). In the memory-less case, the difference in distribution is even more pronounced, albeit the large difference in convergence makes the distributions overall less comparable.

In our setting layer normalization performed best. However, the impact of further increases in state space size seems a promising line of research, since it seems to impact the performance significantly and real-world problems are expected to be equipped with large state spaces. Furthermore, normalization choices influence the arising offer distributions even at similar rates of convergence. Hence, normalization choices should not be taken solely on a technical basis but consideration is necessary that they also affect equilibrium outcomes.

#### State Space and Memory

In Sects. 5.4.1 and 5.4.2 we have shown that larger state spaces do not necessarily lead to better convergence behavior in our setting, at least not in the relatively simple case where state space information is given by past behavior of all agents. It seems, that larger state spaces are more error prone than smaller state spaces.

**Fig. 8:**
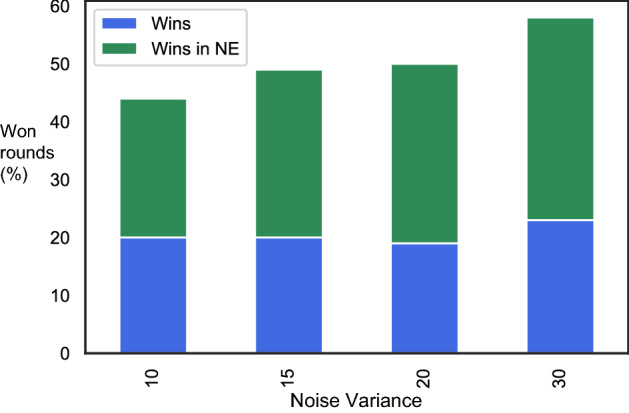
Asymmetric state space memory. *Notes:* The figure depicts the outcome of competition between an agent with state space memory and an agent without. The state space memory contains last rounds offers. The agents are otherwise identical competing in a duopoly. The height of the entire bar (blue and green area) depicts the rate of games, where the agent with memory earned more than its rival (i.e. “won”). Green indicates that the agent with memory wins and the resulting outcome is also a Nash equilibrium. The opposing player has no information on past offers in the state space. However, both players are equipped with a “memory buffer.” The figure illustrates that larger memory in the state space depends on the variance of the noise and is not always advantageous

In order to test this hypothesis more rigorously, we consider a test-case of a classic Bertrand duopoly in which both firms are identical, except that one company includes last rounds offers into its state space and the other not. Figure 8 depicts the results of 100 rounds of competition in such a duopoly. The y-axis plots the ratio of won rounds to total rounds by the agent that has access to the offers of the last round. The x-axis depicts the influence of increasing an agent’s initial exploration rate. Specifically, we set the standard deviation of the initial noise to 10, 15, 20, and 30. The entire bar (blue and green) depicts the percentage of runs, where the agent with memory earns more than its rival (i.e., “wins”). The blue bar shows all cases where the player with memory won and both players are playing a best response, hence have already equilibriated. Hence, if the green bar is above 50%, the availability of memory led to an advantage.

Intuitively, one might believe that more information is a strict advantage and should always benefit an agent. However, Fig. 8 shows that this is not always the case. In our simulations, the agent with no additional information performed better for some parameter settings. Indeed, for weak noise variance of about 10, larger memory is a disadvantage. Interestingly, stronger noise variance seems to counteract this tendency. It can be concluded that the relationship between available state information and algorithmic success is complex. This reemphasizes our findings from Sects. 5.4.1 and 5.4.2 that a larger state space is not necessarily advantageous.

### Qualitative Analysis

**Fig. 9:**
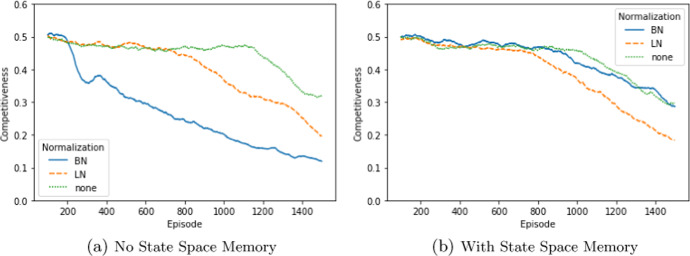
Competitiveness influenced by normalization and memory. *Notes:* To measure competitiveness, we depict the average rate of undercuts per episode from 100 DDPG runs smoothed by a rolling average over 100 episodes. Each plot shows three normalization methods: unnormalized (green), layer normalized (orange), and batch normalized (blue). Panel **a** represents runs without explicit state space memory, while panel **b** allows for state space memory

So far we have tried to remain as quantitative and explicit as possible when assessing the performance of DDPG to derive equilibrium outcomes in uniform price auctions. We complement this assessment with a brief qualitative discussion. The learning progress throughout a run can vary significantly and many characteristics are hard to distill into a single numerical measure. Nonetheless certain differences in learning behavior are striking when different parametrizations are contrasted: In Table 3, we have selected four representative model runs for each of the three normalization schemes. Two runs in each normalization category are memory-less and two have been conducted with single round memory. The chosen runs certainly represent a reoccurring pattern, but nonetheless there are other runs in the same category with distinct appearances. Each category has been run 100 times and we briefly comment on impressions that we had when inspecting these runs. In order to reduce noise, the individual impressions gained from Table 3, we follow up with a statistical evaluation of the offer price development throughout the learning process averaged over all 100 runs with the same normalization and memory choices in Fig. 9. There, we depict the development of the average competitiveness between the algorithms throughout the learning process. This is measured for each individual episode by the percentage of runs that undercut each other in a given episode. Overall, competitiveness starts high and eventually decreases, however there are distinct differences in the speed of decline depending on the normalization scheme and the memory model.

**Table 3:**
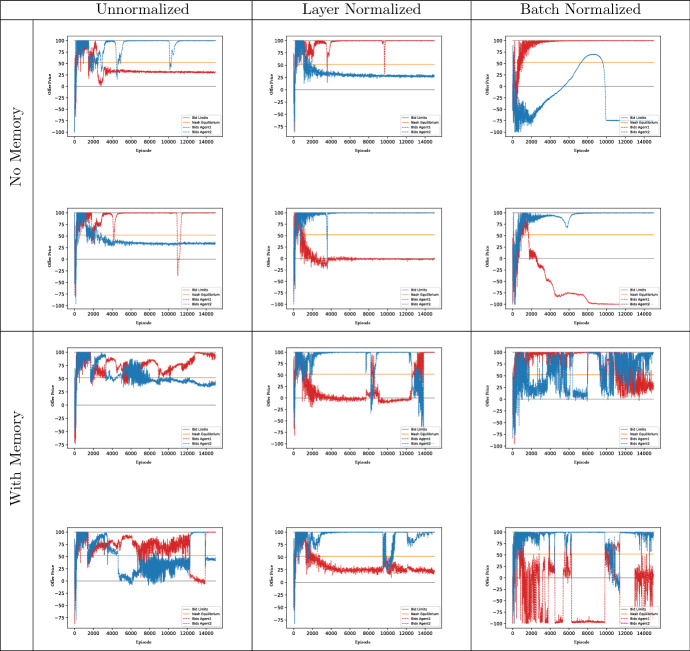
Qualitative depiction of normalization schemes and memory influence on offering behavior. *Notes:* Exemplary selection of 2 out of 100 DDPG runs per normalization methods and memory. Plots in top rows display runs without state space memory (no memory), plots in bottom rows display runs with state space memory (with memory). In the runs with memory, fiercer competition during the learning process seems to be apparent. This assertion is strengthened by Fig. 9’s quantitative evaluation of the average number of undercuts

Overall, the choice of normalization seems to strongly influence behavioral patterns of the agents. We believe that one of the most striking facts is the influence of memory. In Table 3, we see significantly more competition between both participants, whenever we include memory in the state space. The memory less runs tend to settle quickly into a Nash equilibrium and exhibit an overall more static behavior. This seems to come along with a tendency to converge easily.

This observation is confirmed by the statistics from Fig. 9. In all cases, we see considerable more variance in the means of the cases with memory than without, although the range of offers within one standard deviation is only slightly enlarged. This illustrates an overall more unsteady behavior in the non-empty state spaces. The increased variance of means with memory seems to be consistent throughout the different normalization schemes.

Even though we have seen in Sect. 5.4.1 that memory leads to larger sensitivity of convergence towards learning rate choice, we believe that this is not necessarily a sign of bad learning. Indeed, we are inclined to conjecture that the memory allows to know the opponent’s behavior better and thus engage in fiercer competition. From the perspective of market simulation, this may be a more realistic behavior or at least it may be seen as parametrizing some sort of risk-seeking. Hence, we investigate the competitiveness of the two algorithms as a distinct feature of interest.

Initially, it is not clear how to measure competitiveness. We point out that a high variance is not sufficient for ensuring competitveness. For instance, consider the batch-normalized no memory examples depicted in Table 3. There, both low offering players vary there offers considerably, but their price offers never reach the proximity of the high offering player after episode 2000. This is an example of relatively high variance with no competiteveness.

Hence, we propose the following measure of competitiveness. If one player is the high offering player in one turn and the low offering player in the next turn, we count this as a switch. We count the number of switches per episode and divide by the turns per episode, yielding the average switches per episode. This relative measure is normalized to range in [0, 1] with 1 corresponding to a switch in every turn and 0 to never switching. We believe, that this measure clearly captures the notion of competitive behavior, due to being high precisely when opponents undercut each other frequently.

One caveat of the measure may be that it not explicitly considers the magnitude of undercuts, but we still belief it to be informative.

Finally, we smooth the measure by applying a rolling average over a window of 100 episodes. Smoothing is not conceptual, but serves a better visual representation, since the significant variance of the competitiveness measure between episodes leads to indistinguishable graphs.

We depict the development of the algorithms competitiveness measure in Fig. 9. Each color represents a different normalization method. Panel (a) depicts 100 runs without state space memory, while Panel (b) depicts 100 runs with state space memory.

We point out that DDPG is equipped with two possibly interacting types of memory: the replay buffer and a possible state space memory. Hence, results of Panel (a) are not to be understood as memory-less, however they do not include the actions of last round in the state space, but only rely on the statistical representation of the history within the replay buffer of size 50,000. Nonetheless, we see a significant effect of state space memory on the algorithms competitiveness.

First, it has to be noted that we find unormalized runs to be the most competitive ones, while normalization tends to decrease competitiveness overall. Hence, normalization methods can severely affect the resulting outcome.

Generally, state space memory does not strongly impact the development of unnormalized or layer normalized runs. This stand in stark contrast to batch normalized runs, where competitiveness changes completely. While Panel (a) sees batch normalized runs by far the most uncompetitive, in Panel (b) the memory alleviates the normalization effects leaving batch and unnormalized runs almost as competitive.

Contrasting these results with the convergence rates of Fig. 3a, b, we see that while layer normalization leads to perfect convergence and admits consistent behavior regardless of the state space, it comes at the price of a significant decrease in competitiveness and thus essentially makes a behavioral assumption. This is not necessarily a disadvantage, but it illustrates that the choice of the normalization method needs to be in fact a conscious modelling choice.

The decrease in convergence between layer and unnormalized runs is marginal, at least in the empty state space regime that we summarized in Fig. 3a. Indeed, it may be worthwhile to forfeit normalization against common machine learning practices, if competitive behavior is seen as desirable. However, the drop in convergence of unnormalized DDPG runs is significant already in the only slightly enlarged state space of Fig. 3b. It is therefore quite possible that unnormalized DDPG is not robust in complex state spaces.

Another interesting alternative, is the use of batch normalization methods. Although, this normalization method fails both in convergence and competitiveness in Panel (a), it seems to be a sensible choice in Panel (b). If accompanied with state space memory, batch normalization is similarly in competitiveness to unnormalized runs and convergence. Moreover, it is the only method that increased its performance in the larger state space, while all others performed worse. This may indicate a superior scaling of batch normalization to more complex state action spaces. Considering that batch normalization methods stem from computer game learning environments, where each pixel is regarded as individual state it seems at least plausible that this method may work better only in large state spaces. Nonetheless, this can not be fully concluded from the result at hand, but certainly merits deeper investigation to assess this conjecture.

### Replay Buffer & the Limiting Case of the “Ultra-competitive” Benchmark Scenario

In our standard benchmark scenario with demand ${\overline{q}}< D < 2{\overline{q}}$, convergence could be reliably achieved with adequate choices of learning rates and normalization methods. In this case, a replay buffer that was comparably large with up to 50,000 turns memorized was sufficient to attain convergence. We emphasize that in the standard scenario 20,000 episodes were used as a standard run-time. However, algorithms frequently converged already around 5000 episodes.

Classically, Bertrand duopolies are studied in an unconstrained and a symmetric setting. This situation is well known to exhibit a unique equilibrium, where both agents offer their marginal costs. In the unconstrained Bertrand duopoly (i.e., $0< D < {\overline{q}}$), we find DDPG to converge towards both players offering marginal costs as expected. However, the speed of convergence depends crucially on the configuration of the replay buffer. A too large memory buffer leads to slow convergence times. Even extremely long run-times of up to 100,000 episodes did not converge fully, until we reduced the size of the replay buffer. Indeed, in this situation the size of the replay buffer seems absolutely critical. Figure 4, Panel (a)–(c), depict the effect of the memory buffer size on DDPG’s convergence time.

Another parameter that has been proven critical is the noise decay rate. This is less surprising since highly competitive equilibria require sufficient exploration to converge. The “super-competitive” scenario is such a highly competitive equilibrium. Consequently, the effect of the decay rate on the convergence of unconstrained Bertrand games is clearly visible.

#### Replay Buffer Size

We tested replay buffer sizes of 50,000 rounds (approximately 400 Batches), 5000 rounds (approximately 40 Batches), 500 rounds (approximately 4 Batches), 256 rounds (2 Batches), and 129 rounds (1 Batch) each.^12^

Neither very high (50,000 episodes) nor very low choices (129 or 256 episodes) led to good results. Too large memory buffers led to both agents only marginally undercutting each other with a steady but extremely slow decrease in offers over time. Smaller choices of the size of the memory buffer corresponded directly to fast decreases in offers towards the marginal cost equilibrium. However, too small memory buffers (129 or 256 episodes) quickly approached marginal costs but did not necessarily stay within equilibrium. Overall, buffer sizes of 500 converged quickly but yet remained stable. We want to stress that this trend held true both *with and without the inclusion of past actions into the state space*.

We explain this phenomena by the fact that large buffer sizes slow learning. For instance, in the $D\le {\overline{q}}$ case, the equilibrium is only found through long periods of competition. However, initially both algorithms can succeed with relatively large offer prices. Big buffers tend to keep up old experiences for a relatively long time and result in the algorithm decreasing its offer prices slower than with small buffers, as initial successes are remember longer. In contrast, if the memory buffer is not big enough to retain the information that deviations from equilibrium are punished, the algorithm will be unstable as it begins to explore again after convergence to equilibrium. This indicates that replay buffers are indeed an integral part of DDPG and can not be simply left out. Nonetheless, the buffer size should be carefully limited. If convergence is possible, smaller memories are better.

#### Noise & Decay Rate

As its name suggests, deep deterministic policy gradient (DDPG) algorithms employ neural networks that deterministicly output the same values given the same inputs. Outputs only change after training on unseen data. However, novel data would never be explored by a deterministic neural network. Noise introduces randomness into the algorithm, in order to generate novel behavior. Hence, the noise directly controls DDPG’s exploration. In turn, the decay rate controls the fading out of the initially high noise over time that ensures an eventual switch from initial exploration to exploitation. Hence, the decay rate is the most direct correspondence to *Q*-learnings exploitation-exploration parameter.

**Fig. 10:**
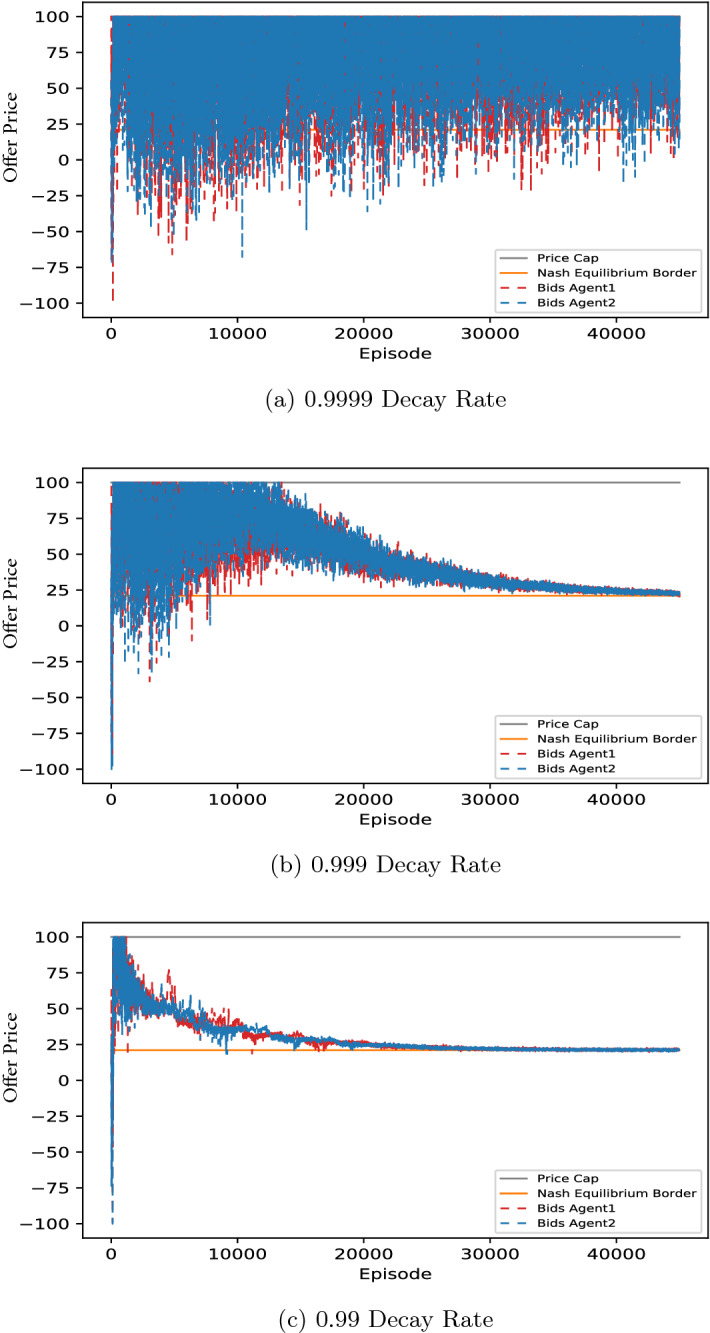
DDPG convergence times of unconstrained bertrand games depending on decay rate size. *Notes:* Figures depict identical DDPG runs with varying decay rates. The decay speed increases from top to bottom. Note that decay rates are multiplicative factors, hence larger numbers correspond to slower decay. The depicted rates are **a** 0.9999, **b** 0.999, and **c** 0.99. Even faster decay rates have a counterproductive effect on convergence times in our setting

We depict the influence of the decay rate on convergence behavior in Fig. 10. The figure shows relatively common behavior similar to many reinforcement algorithms. This is not surprising since the interplay between exploration and exploitation is a shared characteristic of all RL algorithms. Too fast decay rates, such as 0.9, lead to trivial behavior, while slow decay rates such as 0.99999 leads to complete random behavior. In the intermediate regime, convergence is possible with the most-balanced choice of 0.999 leading to the best convergence time in our setting.

### Beyond Duopoly

In this section, we compare oligopolies with 3, 5, and 7 strategic players to the duopoly outcome to understand the algorithmic performance of DDPG when the number of firms increases. For player numbers larger than 2 we lack a theoretical benchmark similar to Proposition 1. We therefore focus on unconstrained symmetric Bertrand scenarios. In that setting, we know that the static Bertrand equilibrium would lead to marginal cost offers. Hence, we are able to analyze the convergence properties of our algorithm in that setting.

We remark that general reinforcement learning is believed to be considerably harder in multi-player settings than in single-player settings (Bu et al. 2008; Foerster et al. 2016). This stems from the fact that every new strategy employed by an agent requires all other agents to recommence learning in response. Insofar, even studying a duopoly is a contribution to the current state-of-the-art in RL. Furthermore, it is generally assumed that convergence times can grow exponentially in the number of players. Hence, every added firm is expected to impact the run-time significantly.

In Sect. 5.6 we studied 100 2-player simulations. We commence by contrasting 100 3-player simulations, 20 5-player simulations, and 20 7-player with respect to their convergence rates after 100,000 iterations. We have not studied higher player numbers since no simulation converged completely in the 7 player case.

**Fig. 11:**
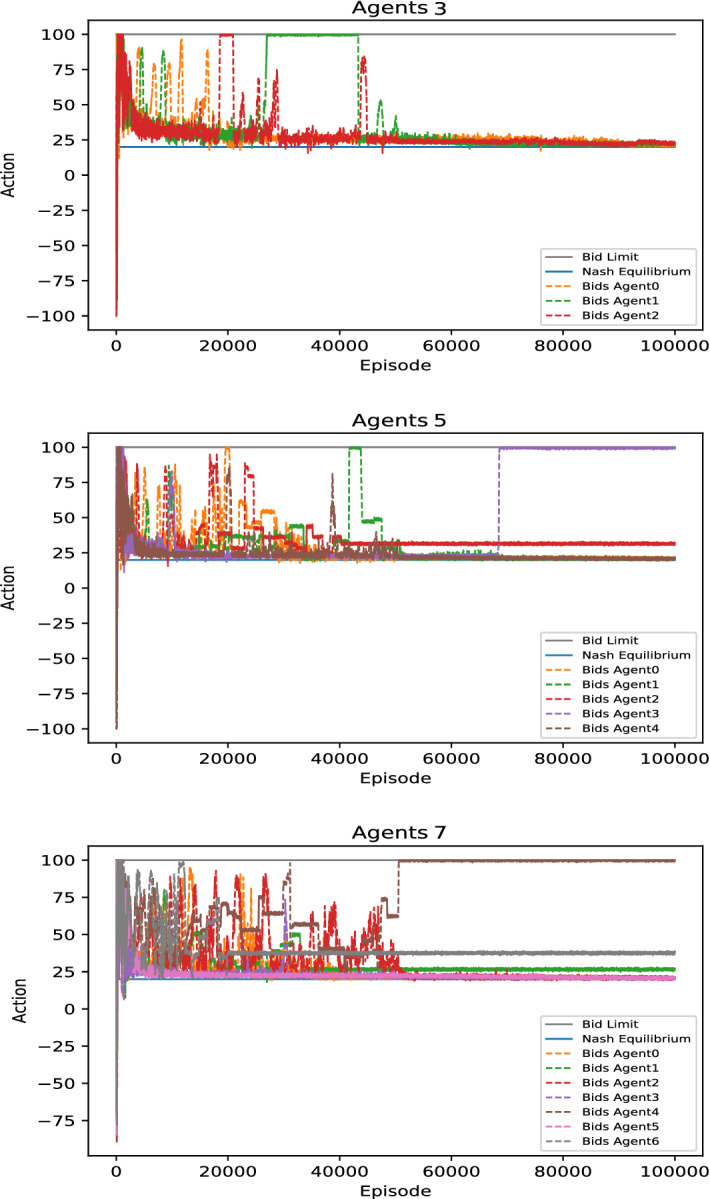
Convergence conditional on the number of players. *Notes:* The figures depicts examples of the development of offers over 100,000 iterations for 3, 5, and 7 strategic players. It is visible that for 5 and 7 players not all players have converged to the Nash equilibrium yet

In agreement with theoretical expectations, the convergence to equilibrium after 100,000 iterations drops with increased player numbers. However, we believe that the results are remarkable nonetheless since they show a much more nuanced scaling behavior than expected. In our setting, 2 or 3 players are indeed tractable within at most 100,000 iterations. We were able to find convergence to equilibrium for 100 out of 100 2-player games, and for 95 out of 100 3-player games. In the 5-player case only 1/20 converged and in the 7-player game we did not find convergence. However, even though after 100,000 iterations full equilibrium was never reached by 7 players (i.e., 7 players simultaneously playing their best response), on average 5 out of 7 players converged to their best response with only 2 players maintaining high volatility in their actions. Hence, there is a clearly visible trend towards the equilibrium present even in the unconverged runs. This is shown by example runs for 3, 5, and 7 agents in Fig. 11.

## Conclusion

Our main contribution is the *adaption of the deep deterministic policy gradient (DDPG)* algorithm to a novel problem domain: *strategic interaction* through a uniform price auction with a continuous action space.^13^

With regard to the neural network design, we closely followed (Lillicrap et al. 2015). Overall, this design seems workable for analyzing competition in auctions. We also remark that Lillicrap’s design seemed like a reasonable starting point, but there is a vast range of possible design choices. However, we believe that refined network designs are an exciting avenue of future research.

We believe and argue that DDPG is a significant step beyond the frequently used *Q*-learning algorithm. The methodological novelty lies in the possibility of allowing fully continuous state and action spaces (as compared to *Q*-learning) and no need for any assumptions of a functional form parametrizing the strategy space of the agents (as compared to policy gradient learning). We have argued that despite frequently being labelled “model-free,” *Q*-learning’s state and action space assumptions indeed require significant modeling assumptions to be justified. Essentially, DDPG’s continuity properties are another step forward towards being truly model-free. Nonetheless, we have pointed out the caveat that certain parametric choices such as memory models and normalization methods do impact agent behavior.

Furthermore, the problem is naturally a multi-agent problem and applying the initially single-agent DDPG to multi-agent settings is in itself a timely endeavour, although there have already been several works pursuing this new line of research. In summary, we believe to have demonstrated that deep reinforcement learning algorithms like DDPG essentially remove the necessity to discretize strategic offering state and action spaces in low feature spaces. This result is most likely generalizable to scenarios with numerous features, however this needs to be ascertained by future work.

We accompany our DDPG implementation with a benchmark framework based on its ability to consistently converge to Nash equilibria. This is only possible through our employment of a scenario (see Sect. 4) where we can analytically derive Nash equilibria. In our case, we consider a duopoly engaged in a uniform price auction of a capacity constrained homogeneous good. We are able to completely characterize the auctions equilibrium strategies. We have performed a number of DDPG market simulations while varying the key learning parameters: *actor learning rate, critic learning rate, state space memory*, and *normalization technique*. Each parametrization has been run 100 times and evaluated with respect to the percentage of strategies that converged to an equilibrium after 15,000 episodes of learning. This allows us to assess the impact of the aforementioned parameters on the learnability of the equilibria.

Despite considerable variance in the results between differing algorithm parametrizations, there is a common trend: Well parametrized DDPG simulations play equilibrium strategies almost always (more than 95% of the runs). Ill-parametrized runs may not converge at all. Nonetheless, our first key finding is that it is possible to reliably find equilibrium strategies with a properly tuned DDPG algorithm. Commonly, the success of deep learning algorithms depends crucially on the correct parametrization. Hence, we give specific advise on how to choose these parameters in Sect. 5.5. We discuss the effects of *explicit state space memory*, *replay buffer memory*, and *normalization method* encountered during our analysis. All showed significant impacts on the algorithm’s behavior. Furthermore, layer normalization clearly performed best in our benchmark, if measured solely in terms of convergence reliability.

Including an *explicit memory* of the last round’s actions as state information had counterintuitive results. Similarly, using a *larger replay buffers* did not necessarily perform better. In contrast, larger replay buffers led to slower convergence times. One might have expected memory to “help” the algorithm and thus converge more easily, but we found the opposite effect. Memory-less algorithms converged in a wider range of learning rates. Nonetheless, both memory-less and algorithms with memory reached convergence rates above 90%, albeit the memory-less ones did so more robustly. This result certainly merits further investigation, however we conjecture two possible reasons for this behavior. First, the large state space of the memory might counteract any informational benefits. This would indicate that one should include state space information only if one is certain that it is vital information. Second, the algorithms with memory appear to compete more proactively. Possibly, awareness of the opponent is necessary to place competitive offers. It may be the case that competition seeking agents explore the strategy space more thoroughly and thus require longer equilibration times. In our opinion, this could be beneficial for market simulations or policy analysis, where one might hope to fully explore the strategy space. Overall, this leads us to conclude that, with respect to algorithmic memory, less seems to be more.

Furthermore, we have to stress the impact of *normalization schemes* on final behavior. Generally, unnormalized runs did perform well too, however the best convergence rates were encountered by the use of so-called layer normalization. Layer normalization tended to produce well-equilibrated, but also relatively static and non-competitive behavioral patterns. This result stood in contrast to batch normalization. Batch normalization showed highly differentiated behavior. While memory-less batch normalization led to the worst convergence rates by far, batch normalization with memory performed well albeit worse than layer normalization with memory, and was the only method that profited from the inclusion of memory. This may be an indication that batch normalization is better suited to handle more complex state spaces; which is also a question for future research. Moreover, it showed an extremely pronounced tendency to compete, experiment, and explore the state space. This property may or may not be desirable, but we certainly see significant behavioral assumptions entering through seemingly inconspicuous normalization methods. Hence, we advise to take this into account.

In summary, we believe to have demonstrated that Nash equilbria in multi-agent uniform price auctions can be found by DDPG simulations. This makes it a promising tool to analyze auctions or to derive informative counterfactuals even in more general settings.

## Data Availability

Not applicable.

## Footnotes

1 Note that the unconstrained static Bertrand equilibrium where players compete in prices can also be formulated in a two-stage setting where players submit offer prices to an auction which will return the award, i.e., the possibility to serve demand at the clearing price, to the player with the lowest offer price or split the demand in case of a tie. Although the two-stage setting seems unnecessarily complicated for these kind of problems, it will become helpful when we transition to more complex strategic interactions between players, e.g., if they are capacity constrained.

2 In some cases, for example, if the only “system-condition” is to ensure that supply equals demand, these problems can be simplified by integrating the equilibrium condition into the first stage. However, for more complicated auction clearing mechanisms, e.g., locational pricing markets (see e.g., Yao et al., 2008; Graf & Wolak, 2020), that is no longer the case.

3 The auction mechanism is equipped with tie-breaking rules that determine what happens if both firms offer at the same price. In our case, both market participants will serve half of the demand in case of a tie.

4 Short-term wholesale electricity markets are typically organized sequentially starting with the day-ahead market and allowing market participants to update their positions until (close to) real-time. For more details, see, e.g., Ito and Reguant (2016), Graf et al. (2020b), or Graf et al. (2021).

5 See for example Reguant (2014), Graf et al. (2020a), or Jha and Leslie (2020) on the importance of these issues.

6 https://github.com/ckrk/bidding_learning.

7 Note, that (7)’s RHS is a proper generalization of its LHS, in the following sense: The LHS is only sensible for discrete values of *a*. In contrast, the RHS approximation of (7) makes sense for continuous *a*, but is equally sensible for discrete *a*. Consequently, both variants are applied within RL. If (7)’s *a* is taken discrete, the algorithm is called “Deep *Q*-Learning (DQN),” while for continuous *a* we call it a “Deep Deterministic Policy Gradient (DDPG)” algorithm.

8 Most of these assumptions can be relaxed easily to model more realistic cases. However, the main purpose of our paper is to test specifications of the algorithms that is why we have chosen an analytically tractable benchmark scenario.

9 An episode corresponds to a single round of submitting offers and subsequent market clearing.

10 Our simulation found that 17 out of 20 runs converged.

11 The decay rate is yet another parameter of the RL algorithm.

12 The choice of low memory buffer sizes are determined as batch size plus one and batch size times two.

13 If players do not have capacity constraints these setting translates to price competition à la Bertrand.

## Code Availability

https://github.com/ckrk/bidding_learning.

## References

[CR1] Adami C, Schossau J, Hintze A (2016). Evolutionary game theory using agent-based methods. Physics of Life Reviews.

[CR2] Aliabadi DE, Kaya M, Şahin G (2017). An agent-based simulation of power generation company behavior in electricity markets under different market-clearing mechanisms. Energy Policy.

[CR3] Andreoni J, Miller JH (1995). Auctions with artificial adaptive agents. Games and Economic Behavior.

[CR4] Asker, J., Fershtman, C., & Pakes, A. (2021). *Artificial intelligence and pricing: The impact of algorithm design*. Technical report, National Bureau of Economic Research. https://www.nber.org/system/files/working_papers/w28535/w28535.pdf

[CR5] Awerbuch, B., Azar, Y., Epstein, A., Mirrokni, V. S., & Skopalik, A. (2008).Fast convergence to nearly optimal solutions in potential games. In *Proceedings of the 9th ACM conference on Electronic commerce*, pp. 264–273. 10.1145/1386790.1386832

[CR6] Ba, J. L., Kiros, J. R., & Hinton, G. E. (2016). Layer normalization. arXiv:1607.06450

[CR7] Blum, A., Hajiaghayi, M., Ligett, K., & Roth, A. (2008) Regret minimization and the price of total anarchy. In *Proceedings of the fortieth annual ACM symposium on Theory of computing*, pp. 373–382. 10.1145/1374376.1374430

[CR8] Boyer CN, Brorsen BW (2014). Implications of a reserve price in an agent-based common-value auction. Computational Economics.

[CR9] Brown, N., Lerer, A., Gross, S., & Sandholm, T. (2019). Deep counterfactual regret minimization. In *International conference on machine learning* (pp. 793–802). PMLR. http://proceedings.mlr.press/v97/brown19b/brown19b.pdf

[CR10] Bu L, Babu R, De Schutter B (2008). A comprehensive survey of multiagent reinforcement learning. IEEE Transactions on Systems, Man, and Cybernetics, Part C (Applications and Reviews).

[CR11] Calvano E, Calzolari G, Denicolò V, Pastorello S (2020). Artificial intelligence, algorithmic pricing, and collusion. American Economic Review.

[CR12] Caoui E (2022). A study of umbrella damages from bid-rigging. The Journal of Law and Economics.

[CR13] Charankevich, H. (2021). *Bid manipulation in open procurement auctions*. Working paper, University of Virginia. https://drive.google.com/file/d/1LoRThIkEjAf-VMnW8yYgRq7Yaqy56PPS

[CR14] Cybenko G (1989). Approximation by superpositions of a sigmoidal function. Mathematics of Control, Signals and Systems.

[CR15] Deissenberg C, Van Der Hoog S, Dawid H (2008). Eurace: A massively parallel agent-based model of the European economy. Applied Mathematics and Computation.

[CR16] Foerster, J., Assael, I. A., de Freitas, N., & Whiteson, S. (2016). Learning to communicate with deep multi-agent reinforcement learning. In *Proceedings of the 30th international conference on neural information processing systems*, p. 29. https://arxiv.org/pdf/1605.06676.pdf

[CR17] Foster DP, Vohra RV (1997). Calibrated learning and correlated equilibrium. Games and Economic Behavior.

[CR18] Fudenberg D, Levine DK (1998). The theory of learning in games.

[CR19] Fudenberg D, Maskin E (1986). The folk theorem in repeated games with discounting or with incomplete information. Econometrica.

[CR20] Fujimoto, S., van Hoof, H., & Meger, D. (2018). Addressing function approximation error in actor-critic methods. *CoRR*, arXiv:1802.09477

[CR21] Graf, C., & Wolak, F. A. (2020). *Measuring the ability to exercise unilateral market power in locational-pricing markets: An application to the Italian electricity market*. Working paper, University of Stanford. https://web.stanford.edu/group/fwolak/cgi-bin/sites/default/files/Measuring in_Locational_Pricing_Markets_Graf_Wolak.pdf

[CR22] Graf, C., Quaglia, F., & Wolak, F. A. (2020a). *Market performance assessment in locational markets with non-convexities*. Working paper, University of Stanford. https://web.stanford.edu/group/fwolak/cgi-bin/sites/default/files/NonConvexBenchmark.pdf

[CR23] Graf, C., Quaglia, F., & Wolak, F. A. (2020b). *Simplified electricity market models with significant intermittent renewable capacity: Evidence from Italy*. NBER Working Papers 27262, National Bureau of Economic Research. http://www.nber.org/papers/w27262

[CR24] Graf C, Quaglia F, Wolak FA (2021). (Machine) learning from COVID-19 lockdown about electricity market performance with a large share of renewables. Journal of Environmental Economics and Management.

[CR25] Greenberg, H. J. (2010). Myths and counterexamples in mathematical programming. *Mathematical programming glossary*. https://glossary.informs.org/myths/CurrentVersion/myths.pdf

[CR26] Guerre E, Perrigne I, Vuong Q (2000). Optimal nonparametric estimation of first-price auctions. Econometrica.

[CR27] Harrison GW (1989). Theory and misbehavior of first-price auctions. The American Economic Review.

[CR28] Hinton GE, Deng L, Yu D, Dahl GE, Mohamed A, Jaitly N, Senior A, Vanhoucke V, Nguyen P, Sainath TN, Kingsbury B (2012). Deep neural networks for acoustic modeling in speech recognition: The shared views of four research groups. IEEE Signal Processing Magazine.

[CR29] Hommes CH (2006). Heterogeneous agent models in economics and finance. Handbook of Computational Economics.

[CR30] Ioffe, S., & Szegedy, C. (2015). Batch normalization: Accelerating deep network training by reducing internal covariate shift. *CoRR*, arXiv:1502.03167

[CR31] Ito K, Reguant M (2016). Sequential markets, market power, and arbitrage. American Economic Review.

[CR32] Jha, A., & Leslie, G. (2020). *Dynamic costs and market power: Rooftop solar penetration in Western Australia*. Technical report, SSRN Working Paper.

[CR33] Kastl J (2011). Discrete bids and empirical inference in divisible good auctions. The Review of Economic Studies.

[CR34] Kingma, D. P., & Ba, J. (2015). Adam: A method for stochastic optimization, 2014. cite arxiv:1412.6980. *Comment: Published as a conference paper at the 3rd International Conference for Learning Representations*, San Diego.

[CR35] Krizhevsky A, Sutskever I, Hinton GE, Pereira F, Burges CJC, Bottou L, Weinberger KQ (2012). Imagenet classification with deep convolutional neural networks. Advances in neural information processing systems.

[CR36] Lago J, Poplavskaya K, Suryanarayana G, De Schutter B (2021). A market framework for grid balancing support through imbalances trading. Renewable and Sustainable Energy Reviews.

[CR37] Lehna M, Hoppmann B, Scholz C, Heinrich R (2022). A Reinforcement Learning approach for the continuous electricity market of Germany: Trading from the perspective of a wind park operator. Energy and AI.

[CR38] Lillicrap, T. P., Hunt, J. J., Pritzel, A., Heess, N., Erez, T., Tassa, Y., Silver, D., & Wierstra, D. (2015). Continuous control with deep reinforcement learning. arXiv:1509.02971

[CR39] Lussange J, Lazarevich I, Bourgeois-Gironde S, Palminteri S, Gutkin B (2021). Modelling stock markets by multi-agent reinforcement learning. Computational Economics.

[CR40] Merlo A, Schotter A (1992). Theory and misbehavior of first-price auctions: Comment. The American Economic Review.

[CR41] Mnih V, Kavukcuoglu K, Silver D, Rusu AA, Veness J, Bellemare MG, Graves A, Riedmiller M, Fidjeland AK, Ostrovski G (2015). Human-level control through deep reinforcement learning. Nature.

[CR42] Noe TH, Rebello M, Wang J (2012). Learning to bid: The design of auctions under uncertainty and adaptation. Games and Economic Behavior.

[CR43] Reguant M (2014). Complementary bidding mechanisms and startup costs in electricity markets. Review of Economic Studies.

[CR44] Roughgarden T (2010). Algorithmic game theory. Communications of the ACM.

[CR45] Schrittwieser J, Antonoglou I, Hubert T, Simonyan K, Sifre L, Schmitt S, Guez A, Lockhart E, Hassabis D, Graepel T, Lillicrap T, Silver D (2020). Mastering Atari, Go, chess and shogi by planning with a learned model. Nature.

[CR46] Schuurmans, D., & Zinkevich, M. A. (2016). Deep learning games. In *Advances in neural information processing systems*, pp. 1678–1686. https://papers.nips.cc/paper/2016/file/c4015b7f368e6b4871809f49debe0579-Paper.pdf

[CR47] Silver D, Huang A, Maddison CJ, Guez A, Sifre L, Van Den Driessche G, Schrittwieser J, Antonoglou I, Panneershelvam V, Lanctot M (2016). Mastering the game of Go with deep neural networks and tree search. Nature.

[CR48] Sirignano J, Cont R (2019). Universal features of price formation in financial markets: Perspectives from deep learning. Quantitative Finance.

[CR49] Spooner, T., Fearnley, J., Savani, R., & Koukorinis, A. (2018). Market making via reinforcement learning. In *Proceedings of the 17th AAMAS*, pp. 434–442.

[CR50] Sutton RS, Barto AG (2018). Reinforcement learning: An introduction.

[CR51] Tesfatsion L, Judd KL (2006). Handbook of computational economics: agent-based computational economics.

[CR52] Thurber MC, Davis TL, Wolak FA (2015). Simulating the interaction of a renewable portfolio standard with electricity and carbon markets. The Electricity Journal.

[CR53] van Hasselt HP, Guez A, Hessel M, Mnih V, Silver D (2016). Learning values across many orders of magnitude. Advances in Neural Information Processing Systems.

[CR54] Viehmann J, Lorenczik S, Malischek R (2021). Multi-unit multiple bid auctions in balancing markets: An agent-based Q-learning approach. Energy Economics.

[CR55] Vinyals O, Babuschkin I, Czarnecki WM, Mathieu M, Dudzik A, Chung J, Choi DH, Powell R, Ewalds T, Georgiev P (2019). Grandmaster level in StarCraft II using multi-agent reinforcement learning. Nature.

[CR56] Viossat Y, Zapechelnyuk A (2013). No-regret dynamics and fictitious play. Journal of Economic Theory.

[CR57] Watkins, C. J. C. H. (1989).*Learning from delayed rewards*. Ph.D. thesis, University of Cambridge.

[CR58] Yao J, Adler I, Oren SS (2008). Modeling and computing two-settlement oligopolistic equilibrium in a congested electricity network. Operations Research.

[CR59] Yi, H. (2018). Deep deterministic policy gradient for autonomous vehicle driving. In *Proceedings on the International Conference on Artificial Intelligence (ICAI)*, pp. 191–194.

[CR60] Zhang Z, Chen J, Chen Z, Li W (2021). Asynchronous episodic deep deterministic policy gradient: Toward continuous control in computationally complex environments. IEEE Transactions on Cybernetics.

